# Five-year survival with neoadjuvant therapy in melanoma: updated pooled analysis from the International Neoadjuvant Melanoma Consortium (INMC)

**DOI:** 10.1038/s41591-026-04677-z

**Published:** 2026-10-01

**Authors:** Georgina V. Long, Inês Pires da Silva, Kristen A. Perry, Jurriaan Romano, Serigne N. Lo, Piyush Grover, Paolo A. Ascierto, Rodabe N. Amaria, Tina J. Hieken, Caroline Robert, Lucie Heinzerling, Shahneen Sandhu, Milton Barros, Tara C. Mitchell, Zeynep Eroglu, Igor V. Samoylenko, Lukas Flatz, Céleste Lebbé, Douglas B. Johnson, Piotr Rutkowski, Eva Muñoz-Couselo, Omid Hamid, Claudia Trojaniello, Domenico Mallardo, Matthew S. Block, Naima Hammoudi, Naima Benannoune, Dirk Tomsitz, Yang Wang, Cigdem Dinckal, Angelina Kuzmenko, Andrea Forschner, Matteo S. Carlino, Mathilde Jalving, Michael A. Postow, P. Francesco Ferrucci, Ellen Kapiteijn, Astrid A. M. van der Veldt, Karijn P. M. Suijkerbuijk, Laetitia da Meda, Hannah Burnette, Anna Czarnekca, Inderjit Mehmi, Geke Hospers, Sarah Lochrin, Alexander C. J. van Akkooi, Hussein A. Tawbi, Elizabeth M. Burton, Michael T. Tetzlaff, Bart A. van de Wiel, Robert V. Rawson, Christian U. Blank, Alexander M. Menzies

**Affiliations:** 1 Melanoma Institute Australia, The University of Sydney, Sydney, New South Wales Australia; 2 Faculty of Health and Medicine, The University of Sydney, Sydney, New South Wales Australia; 3 Charles Perkins Centre, The University of Sydney, Sydney, New South Wales Australia; 4 Royal North Shore and Mater Hospitals, Sydney, New South Wales Australia; 5 Champalimaud Foundation, Lisbon, Portugal; 6 Tygat Institute for Intestinal and Liver Research, Amsterdam UMC, Amsterdam, The Netherlands; 7 Fiona Stanley Hospital, University of Western Australia, Perth, Western Australia Australia; 8 Università degli Studi di Napoli ‘Federico II’, Naples, Italy; 9 Istituto Nazionale Tumori IRCCS Fondazione ‘G. Pascale’, Naples, Italy; 10 University of Texas MD Anderson Cancer Center, Houston, TX USA; 11 Mayo Clinic Comprehensive Cancer Center, Rochester, MN USA; 12 Gustave Roussy, Département de Médecine Oncologique, Université Paris-Saclay, Villejuif, France; 13 LMU University Hospital, Munich, Germany; 14 Peter MacCallum Cancer Centre, Melbourne, Victoria Australia; 15 Centro de Referência em Tumores Cutâneos, AC Camargo Cancer Center, São Paulo, Brazil; 16 Perelman School of Medicine, University of Pennsylvania, Philadelphia, PA USA; 17 H. Lee Moffitt Cancer Center & Research Institute, Tampa, FL USA; 18 FSBI ‘N. N. Blokhin National Medical Research Center of Oncology’ of the Ministry of Health of the Russian Federation, Moscow, Russian Federation; 19 University of Tübingen, Tübingen, Germany; 20 Université Paris Cite, AP-HP Dermato-oncology and CIC, Cancer Institute APHP.nord Paris cité, INSERM U1342—Equipe—CNRS EMR8000, Saint Louis Hospital, Paris, France; 21 Vanderbilt University Medical Center, Nashville, TN USA; 22 Maria Sklodowska-Curie National Research Institute of Oncology, Warsaw, Poland; 23 Vall d’Hebrón Hospital and Vall d’Hebrón Institute of Oncology (VHIO), Barcelona, Spain; 24 The Angeles Clinic and Research Institute, Los Angeles, CA USA; 25 Westmead and Blacktown Hospitals, Sydney, New South Wales Australia; 26 University Medical Center Groningen, Groningen, The Netherlands; 27 Memorial Sloan Kettering Cancer Center and Weill Cornell Medical College, New York, NY USA; 28 European Institute of Oncology, IRCCS Milan, Milan, Italy; 29 Leiden University Medical Center, Leiden, The Netherlands; 30 Erasmus Medical Center, Rotterdam, The Netherlands; 31 University Medical Center Utrecht, Utrecht University, Utrecht, The Netherlands; 32 Royal Prince Alfred Hospital, Sydney, New South Wales Australia; 33 University of California San Francisco, San Francisco, CA USA; 34 Netherlands Cancer Institute, Amsterdam, The Netherlands; 35 NSW Health Pathology, Sydney, New South Wales Australia; 36 University Clinic Regensburg, Regensburg, Germany; 37 European Institute of Oncology, Milan, Italy

**Keywords:** Melanoma, Immunotherapy

## Abstract

Neoadjuvant immune checkpoint inhibitors (ICIs) are standard of care for resectable stage IIIB–D melanoma, but knowledge gaps remain regarding optimal treatment type, long-term survival, postsurgical management and clinical, histopathological or molecular subgroups. We analyzed 1,038 patients treated with neoadjuvant ICIs (*n* = 735), BRAF and MEK inhibitors (BRAF/MEKis; *n* = 119) or ICI plus any targeted therapy (ICI + TT; *n* = 184) from 26 international sites in both trial (755, 72.7%) and nontrial (283, 27.3%) settings. ICI regimens included anti-programmed cell death protein 1 (PD-1) alone (PD-1 alone; *n* = 242) and anti-PD-1 plus other immuno-oncology agent(s) (PD-1 + IO; *n* = 489). Outcomes included major pathological response (MPR), recurrence-free survival (RFS) and overall survival (OS). An MPR was achieved in 57.8% of patients with ICIs, 51.4% with BRAF/MEKis and 44.6% with ICI + TT. PD-1 + IO elicited higher MPR rates than PD-1 alone (61.4% versus 49.5%). The 5-year RFS rate was 61.0% with PD-1 alone, 73.9% with PD-1 + IO, 37.4% with BRAF/MEKis and 76.4% with ICI + TT; the 5-year OS rates were 83.3%, 87.5%, 69.8% and 83.9%, respectively. ICI-treated patients with MPR had 5-year OS rates of 98.8% with PD-1 alone and 97.9% with PD-1 + IO and gained no benefit from continuing ICIs into the adjuvant setting. Neoadjuvant ICIs were active in *BRAF*-mutant melanoma (MPR, 46.3%), acral melanoma (38.1%), in-transit metastases (66.7%) and oligometastases (41.2%), although subgroup sizes and relative effectiveness varied. Survival outcomes were excellent with neoadjuvant ICIs, particularly in patients achieving MPR, but poor with BRAF/MEKis and mixed with ICI + TT. Predictive biomarker-driven trials, effective adjuvant strategies and subgroup-specific research are needed for nonresponders.

## Abstract (web summary)

An updated pooled analysis of 3-year and 5-year survival and other outcome data of 1,038 patients with melanoma treated with neoadjuvant immune checkpoint inhibitors (ICIs), BRAF and MEK inhibitors (BRAF/MEKis), or a combination of ICIs and targeted therapies shows that ICI as monotherapy or in ICI combinations are superior to BRAF/MEKis and patients with major pathological responses have better long-term survival.

## Main

Historically, patients with clinically detected, stage III melanoma had a very high risk of recurrence and death, even after definitive surgery^1,2^. In the early to mid-2010s, immune checkpoint inhibitors (ICIs) and BRAF and MEK inhibitors (BRAF/MEKis) emerged as the first effective systemic therapies for advanced melanoma^3–5^. Subsequent trials found that ICIs and BRAF/MEKis were also effective as postsurgical adjuvant therapies in clinically detected, resectable, stage III melanoma, roughly halving the risk of recurrence^6–8^; to date, however, no overall survival (OS) benefit has been reported.

Neoadjuvant immunotherapy, which involves treating resectable tumors with ICIs before rather than after definitive surgery, has now superseded the adjuvant approach in melanoma. Preclinical and translational studies suggest that exposing the whole tumor and intact draining nodal basin to ICIs may generate a better immune response toward the tumor than targeting residual microscopic disease after surgery^9–12^. Neoadjuvant anti-programmed cell death protein 1 (PD-1) alone and neoadjuvant anti-PD-1 plus anti-CTLA-4 each markedly improved event-free survival (EFS) compared with adjuvant anti-PD-1 (hazard ratios (HRs) 0.52 and 0.32, respectively)^13,14^. The magnitude of benefit reported at 1–3 years of follow-up^13–21^, supported by over a decade of experience with ICIs in the advanced and adjuvant settings, enabled rapid international recommendation of neoadjuvant immunotherapy in clinical practice guidelines^18–21^.

As neoadjuvant therapy enters routine care, it is critical that clinical and translational research efforts continue in order to solidify and expand the current evidence base. This requires larger cohorts with longer follow-up to validate and monitor the durability of the response. Many practical clinical questions remain unanswered: Which neoadjuvant ICIs should be used? Is there a role for BRAF/MEKis and other targeted therapies? Should treatment be continued postsurgery and, if so, which treatment and for which patients? What is the impact of a *BRAF* mutation on response? How active is neoadjuvant therapy in acral melanomas? Can neoadjuvant therapy be considered for patients with resectable in-transit lesions or oligometastatic stage IV melanoma? It is also unclear whether therapeutic surgery can be omitted in some patients where a complete pathological response (pCR) or major pathological response (MPR) is generalized from a smaller resection specimen or imaging.

In 2021, the International Neoadjuvant Melanoma Consortium (INMC) published a pooled analysis of 6 early phase trials^17,22–26^ of neoadjuvant ICIs and BRAF/MEKis in 192 patients with resectable stage IIIB–D melanoma^27^. The analysis reported 2-year EFS rates ≥90% in those achieving any pathological response to neoadjuvant anti-PD-1 alone or anti-PD-1 + anti-CTLA-4. Since then, the number of patients treated with neoadjuvant therapy has grown and the median follow-up of the original pooled trial cohort is >5 years.

Here we report an updated pooled analysis of pathological response and survival in an expanded cohort of 1,038 patients with cutaneous, clinically detected, resectable, stage IIIB–IV melanoma treated with neoadjuvant ICIs, BRAF/MEKis or ICIs plus any targeted therapy (TT). Across these regimens, we defined the effectiveness of treatment and established the long-term prognostic value of pathological response. Within the ICI-treated cohort, we assessed the impact of continuing drug treatment postsurgery, the activity of neoadjuvant therapy in molecular, histopathological and clinical subgroups and how clinical trial benchmarks are translating into off-trial settings.

## Results

### Patients and treatment

From 16 November 2023 to 29 October 2024, data from 1,038 patients with cutaneous (including acral), clinically detected, resectable, stage IIIB–IV melanoma treated with neoadjuvant systemic therapy was collected from 26 international cancer research centers and university teaching hospitals (Supplementary Table 1; for the first patient, the first dose was 9 August 2011 and for the last patient, the first dose was 12 August 2024). There were 755 (72.7%) clinical trial patients and 283 (27.3%) treated outside a trial setting. Patients received neoadjuvant ICIs (*n* = 735, 70.8%), BRAF/MEKis (*n* = 119, 11.5%) or ICI + TT (*n* = 184, 17.7%). The ICI cohort included 242 (23.3%) patients treated with anti-PD-1 alone (PD-1 alone) and 489 (47.1%) with a combination of anti-PD-1 and any other immuno-oncology agent(s) (PD-1 + IO). Combination ICI regimens included 386 (37.2%) patients treated with anti-PD-1 plus anti-CTLA-4 (PD-1 + CTLA-4), 58 (5.6%) with anti-PD-1 plus anti-LAG3 (PD-1 + LAG3) and 45 (4.3%) with anti-PD-1 plus other IO (PD-1 + other IO). A complete list of neoadjuvant regimens is given in Supplementary Table 2 and clinical trials are listed in Supplementary Table 3.

Baseline characteristics are shown in Table 1. The median age was 60 years (interquartile range (IQR) 49–69 years) and most patients were male (male, 61.8%; female, 38.2%). *BRAF* V600 mutations were found in 49.1% (510). There were 608 (58.6%) cutaneous primaries, 29 (2.8%) acral primaries and 210 (20.2%) occult primaries. A total of 1,003 (96.6%) patients had stage III melanoma, including 855 (82.4%) with nodal disease alone, 74 (7.1%) with in-transit metastases alone and another 74 (7.1%) with nodal plus in-transits; 35 (3.4%) had resectable oligometastatic stage IV melanoma. Age, sex and stage were balanced between treatment groups. The BRAF/MEKi cohort had a higher proportion of substage IIIC patients and higher median sum of diameters (SOD) per computed tomography (CT) scan than the ICI and ICI + TT cohorts.

**Table 1:** Baseline characteristics and disposition

		ICIs^a^ (*n* = 735)		BRAF/MEKis (*n* = 119)	ICI + TT^a^ (*n* = 184)	Overall (*n* = 1,038)
	All (*n* = 735)	PD-1 alone (*n* = 242)	PD-1 + IO^b^ (*n* = 489)			
**Characteristics**						
**Age, years, median (IQR)**	61 (50–70)	62 (52–70)	59 (48–69)	56 (47–66)	59 (47–70)	60 (49–69)
**Sex,** ***n*** **(%)**						
Female	277 (37.7)	101 (41.7)	174 (35.6)	52 (43.7)	68 (37.0)	397 (38.2)
Male	458 (62.3)	141 (58.3)	315 (64.4)	67 (56.3)	116 (63.0)	641 (61.8)
**Primary site,** ***n*** **(%)**						
Extremity	189 (25.7)	76 (31.4)	112 (22.9)	42 (35.3)	30 (16.3)	261 (25.1)
Trunk	159 (21.6)	58 (24.0)	100 (20.4)	31 (26.1)	33 (17.9)	223 (21.5)
Head and neck	109 (14.8)	38 (15.7)	71 (14.5)	8 (6.7)	7 (3.8)	124 (11.9)
Acral	26 (3.5)	9 (3.7)	15 (3.1)	0 (0)	3 (1.6)	29 (2.8)
Occult	154 (21.0)	53 (21.9)	101 (20.7)	20 (16.8)	36 (19.6)	210 (20.2)
***BRAF*** **mutation status,** ***n*** **(%)**						
Wild-type	363 (49.4)	113 (46.7)	248 (50.7)	0 (0)	73 (39.7)	436 (42.0)
V600	283 (38.5)	90 (37.2)	192 (39.3)	117 (98.3)	110 (59.8)	510 (49.1)
Non-V600^c^	26 (3.5)	6 (2.5)	20 (4.1)	2 (1.7)	1 (0.5)	29 (2.8)
**Baseline disease,** ***n*** **(%)**						
Nodal only	626 (85.2)	196 (81.0)	428 (87.5)	79 (66.4)	150 (81.5)	855 (82.4)
In-transits only^d^	38 (5.2)	18 (7.4)	20 (4.1)	27 (22.7)	9 (4.9)	74 (7.1)
Nodal plus in-transits^d^	54 (7.3)	25 (10.3)	27 (5.5)	9 (7.6)	11 (6.0)	74 (7.1)
**Nodal site,** ***n*** **(%)**						
Axilla	283 (38.5)	81 (33.5)	202 (41.3)	38 (31.9)	82 (44.6)	403 (38.8)
Neck	177 (24.1)	54 (22.3)	123 (25.2)	11 (9.2)	31 (16.8)	219 (21.1)
Groin	223 (30.3)	85 (35.1)	135 (27.6)	45 (37.8)	66 (35.9)	334 (32.2)
**AJCC 8th edn, stage,** ***n*** **(%)**						
III	718 (97.7)	239 (98.8)	475 (97.1)	115 (96.6)	170 (92.4)	1,003 (96.6)
IIIB	333 (45.3)	90 (37.2)	242 (49.5)	29 (24.4)	77 (41.8)	439 (42.3)
IIIC	290 (39.5)	104 (43.0)	184 (37.6)	60 (50.4)	80 (43.5)	430 (41.4)
IIID	15 (2.0)	3 (1.2)	11 (2.2)	4 (3.4)	7 (3.8)	26 (2.5)
IIIB–D NOS^e^	80 (10.9)	42 (17.4)	38 (7.8)	21 (17.6)	6 (3.3)	107 (10.3)
IV	17 (2.3)	3 (1.2)	14 (2.9)	4 (3.4)	14 (7.6)	35 (3.4)
**SOD, mm, median (IQR)**	25 (17–37)	24 (16–40)	25 (18–35)	42 (22–61)	24 (17–38)	25 (18–39)
**SUV**_**max**_**, value, median (IQR)**	13 (8–20)	14 (8–18)	12 (7–22)	15 (11–24)	16 (9–27)	14 (9–22)
**Disposition**						
**Treatment setting,** ***n*** **(%)**						
Trial	463 (63.0)	87 (36.0)	376 (76.9)	112 (94.1)	180 (97.8)	755 (72.7)
Nontrial	272 (37.0)	155 (64.0)	113 (23.1)	7 (5.9)	4 (2.2)	283 (27.3)
**Neoadjuvant therapy**						
Duration, weeks, median (IQR)	7 (6–9)	8 (6–10)	7 (6–9)	9 (8–11)	8 (7–11)	8 (6–10)
Treatment course, *n* (%)						
Completed	650 (88.4)	226 (93.4)	421 (86.1)	111 (93.3)	164 (89.1)	925 (89.1)
Ceased early	76 (10.3)	15 (6.2)	60 (12.3)	8 (6.7)	20 (10.9)	104 (10.0)
**Surgery,** ***n*** **(%)**						
Yes^f^	692 (94.1)	214 (88.4)	476 (97.3)	115 (96.6)	174 (94.6)	981 (94.5)
No	41 (5.6)	27 (11.2)	12 (2.5)	4 (3.4)	10 (5.4)	55 (5.3)
**Adjuvant therapy,**^**g**^ ***n*** **(%)**						
Nil	283 (38.5)	27 (11.2)	255 (52.1)	4 (3.4)	7 (3.8)	294 (28.3)
ICIs	364 (49.5)	177 (73.1)	186 (38.0)	26 (21.8)	156 (84.8)	546 (52.6)
BRAF/MEKis	46 (6.3)	10 (4.1)	36 (7.4)	85 (71.4)	10 (5.4)	141 (13.6)

Baseline characteristics by neoadjuvant regimen. Missing data have been omitted from the table, hence the categorical variables may not sum to 100%. SUV_max_ included 299 patients. Age is at the start of neoadjuvant treatment. Four patients were treated with CTLA-4 alone, hence PD-1 alone and PD-1 + IO may not sum to total ICIs. Indented characteristics or disposition factors are subsets of the preceding, but all percentages are calculated using the denominator in the column header.

^a^PD-1 includes both anti-PD-1 and anti-PD-L1 agents.

^b^IO includes any immuno-oncology agent(s), including other ICIs.

^c^Non-V600 *BRAF* mutations included D594N (*n* = 2), G464V (*n* = 1), G466E (*n* = 1), G469A (*n* = 1), G469K (*n* = 1), G469R (*n* = 2), G469V (*n* = 1), K601E (*n* = 5), L597K (*n* = 1), L597R (*n* = 1), L597S (*n* = 3), other unknown (*n* = 7) and S467L (*n* = 2).

^d^In-transits includes both in-transit and satellite metastases.

^e^Stage IIIB–D patients where AJCC 8th edn substage is unknown.

^f^In 23 cases there was evidence of residual tumor after surgery (ICIs, *n* = 18; BRAF/MEKis, *n* = 1; ICI + TT, *n* = 4).

^g^Systemic drug treatments only.

AJCC, American Joint Committee on Cancer; NOS, not otherwise specified; SUV_max_, maximum standard uptake value.

The median follow-up from the first dose of treatment was 2.6 years (95% CI 2.4–2.9 years). All patients received at least one dose of neoadjuvant therapy, with median treatment duration ranging from 7 weeks to 9 weeks (Table 1). Some 981 (94.5%) patients underwent surgery. Over half (687, 66.2%) of patients received adjuvant drug therapy, including 497 (47.9%) who received adjuvant PD-1 alone, 49 (4.7%) who received adjuvant PD-1 + IO and 141 (13.6%) who received adjuvant BRAF/MEKis. Reasons for surgical omission are found in Extended Data Table 1.

### Pathological response

Pathological response was available for 959 patients (Table 2 and Extended Data Table 2) and was scored by percentage of residual viable tumor in the tumor bed, per the 2018 INMC criteria^28^. MPRs (pCR or near-pCR) occurred in 394 patients treated with ICIs (57.8%, 95% CI 54.1–61.5%), 57 treated with BRAF/MEKis (51.4%, 95% CI 41.7–61.0%) and 74 treated with ICI + TT (44.6%, 95% CI 36.6–52.2%). The pCR rate was 46.8% with ICIs, 38.7% with BRAF/MEKis and 30.7% with ICI + TT; pathological nonresponse (pNR) rates were 30.5%, 26.1% and 38.0%, respectively. Rates of near-pCR (11.7%) and pathological partial response (pPR) (14.0%) were low across the cohort. MPR was significantly associated with treatment type (PD-1 alone, PD-1 + IO, BRAF/MEKis or ICI + TT) after adjusting for potential confounders (Extended Data Table 3).

**Table 2:** Pathological response and survival outcomes

	ICIs^a^ (*n* = 735)			BRAF/MEKis (*n* = 119)	ICI + TT^a^ (*n* = 184)	Overall (*n* = 1,038)
	All (*n* = 735)	PD-1 alone (*n* = 242)	PD-1 + IO^b^ (*n* = 489)			
**Median follow-up,** **years (range)**	2.5 (0.1–11.0)	1.3 (0.1–8.9)	3.0 (0.2–7.4)	4.7 (0.5–9.6)	2.4 (0.3–7.2)	2.6 (0.1–11.0)
**Pathological response**						
*N*	682	208	472	111	166	959
MPR, *n* (%) (95% CI)	394 (57.8) (54.0–61.5)	103 (49.5) (42.5–56.5)	290 (61.4) (56.9–65.9)	57 (51.4) (41.7–61.0)	74 (44.6) (36.9–52.5)	525 (54.7) (51.5–57.9)
pCR	319 (46.8)	84 (40.4)	234 (49.6)	43 (38.7)	51 (30.7)	413 (43.1)
Near-pCR	75 (11.0)	19 (9.1)	56 (11.9)	14 (12.6)	23 (13.9)	112 (11.7)
pPR, *n* (%)	80 (11.7)	28 (13.5)	52 (11.0)	25 (22.5)	29 (17.5)	134 (14.0)
pNR, *n* (%)	208 (30.5)	77 (37.0)	130 (27.5)	29 (26.1)	63 (38.0)	300 (31.3)
**Recurrence-free survival**						
*N* at risk	690	205	475	115	174	979
*n* (%)	160 (23.2)	54 (26.3)	104 (21.9)	67 (58.3)	39 (22.4)	266 (27.2)
Median, months (95% CI)	NR (NR–NR)	NR (60.2–NR)	NR (NR–NR)	22.3 (15.6–54.0)	NR (63.8–NR)	NR (NR–NR)
At 3 years, % (95% CI)	74.0 (70.2–77.9)	67.7 (59.8–76.7)	76.3 (72.1–80.7)	40.7 (32.1–51.5)	76.4 (69.4–84.1)	69.7 (66.4–73.1)
MPR	93.1 (90.2–96.1)	95.6 (89.2–100)	92.8 (89.7–96.1)	60.4 (48.4–75.5)	89.6 (81.5–98.5)	88.2 (85.0–91.5)
pCR	94.1 (91.3–97.1)	100 (100–100)	93.2 (89.7–96.7)	58.4 (44.8–75.9)	91.8 (84.3–99.9)	88.7 (85.2–92.4)
Near-pCR	89.5 (81.8–97.9)	77.4 (52.6–100)	91.8 (84.5–99.9)	70.1 (49.4–99.6)	85.7 (68.3–100)	86.4 (79.3–94.1)
pPR	81.1 (71.8–91.7)	77.6 (61.8–97.4)	82.9 (71.8–95.7)	20.5 (9.0–46.9)	86.2 (74.5–99.7)	68.7 (60.4–78.2)
pNR	38.9 (31.7–47.6)	35.2 (23.9–51.8)	39.8 (31.1–50.8)	20.5 (8.8–47.8)	54.4 (41.0–72.2)	39.9 (3.4–47.2)
At 5 years, % (95% CI)	70.6 (66.3–75.2)	61.0 (51.4–72.4)	73.9 (69.3–78.9)	37.4 (28.7–48.7)	76.4 (69.4–84.1)	66.5 (62.8–70.4)
MPR	91.2 (87.4–95.1)	79.6 (61.3–100)	92.8 (89.7–96.1)	57.4 (45.0–73.3)	89.6 (81.5–98.5)	85.6 (81.4–90.0)
pCR	92.7 (88.8–96.8)	88.9 (70.6–100)	93.2 (89.7–96.7)	54.7 (40.8–73.3)	91.8 (84.3–99.8)	86.0 (80.9–91.3)
Near-pCR	86.4 (77.1–96.8)	51.6 (21.2–100)	91.8 (84.4–99.9)	70.1 (49.4–99.6)	NR	84.1 (76.1–93.0)
pPR	73.0 (60.0–88.8)	77.6 (61.8–97.3)	68.5 (49.8–94.2)	20.5 (9.0–46.8)	86.2 (74.5–99.7)	63.9 (54.2–75.5)
pNR	35.2 (27.8–44.4)	32.0 (20.8–49.1)	36.3 (27.5–47.8)	13.7 (4.3–43.8)	54.4 (41.0–72.2)	36.3 (29.9–44.0)
**Overall survival**						
*N* at risk	735	242	489	119	184	1,038
*n* (%)	68 (9.3)	25 (10.3)	42 (8.6)	27 (22.7)	22 (12.0)	117 (11.3)
Median, months	NR (92.5–NR)	NR (NR–NR)	NR (NR–NR)	NR (108.6–NR)	NR (NR–NR)	NR (108.6–NR)
At 3 years, % (95% CI)	87.4 (84.5–90.5)	83.3 (76.9–90.2)	88.7 (85.4–92.2)	82.0 (74.4–90.3)	83.9 (77.4–90.9)	86.0 (83.4–88.7)
MPR	98.0 (96.4–99.6)	98.8 (96.4–100)	97.9 (96.1–99.8)	90.0 (81.9–98.8)	96.9 (92.7–100)	96.6 (94.7–98.5)
pCR	98.4 (96.8–100)	100 (100–100)	98.0 (96.0–99.9)	89.5 (80.3–99.9)	98.0 (94.3–100)	96.9 (94.9–99.0)
Near-pCR	96.6 (92.1–100)	92.9 (80.3–100)	97.8 (93.6–100)	91.7 (77.3–100)	94.4 (84.4–100)	95.4 (91.0–99.9)
pPR	88.3 (80.3–97.1)	85.0 (70.5–100)	89.6 (80.1–100)	79.7 (61.0–100)	89.3 (78.5–100)	86.9 (80.2–94.1)
pNR	72.9 (65.5–81.1)	74.3 (62.6–88.2)	71.4 (62.1–82.1)	63.2 (46.3–86.3)	69.1 (55.3–86.4)	70.8 (64.5–77.7)
At 5 years, % (95% CI)	86.5 (83.3–89.8)	83.3 (76.9–90.2)	87.5 (83.7–91.4)	69.8 (60.2–80.9)	83.9 (77.4–90.9)	82.7 (79.5–86.0)
MPR	98.0 (96.4–99.6)	98.8 (96.4–100)	97.9 (96.1–99.8)	84.2 (73.8–96.0)	96.9 (92.7–100)	95.1 (92.4–97.9)
pCR	98.4 (96.8–100)	100 (100–100)	98.0 (96.0–100)	86.0 (75.1–98.4)	98.0 (94.3–100)	95.9 (93.0–98.8)
Near-pCR	96.6 (92.1–100)	92.9 (80.3–100)	97.8 (93.6–100)	76.4 (51.4–100)	94.4 (84.4–100)	92.7 (86.3–99.7)
pPR	84.1 (73.5–96.3)	85.0 (70.5–100)	82.1 (67.0–100)	59.8 (36.9–96.7)	89.3 (78.5–100)	77.8 (66.9–90.4)
pNR	71.6 (64.0–80.1)	74.3 (62.6–88.2)	69.4 (59.7–80.7)	43.8 (27.2–70.5)	69.1 (55.3–86.4)	66.6 (59.8–74.1)

Key efficacy outcomes by neoadjuvant regimen. *N* = number of patients for whom the necessary data were available; *n* = number of patients with the outcome (pathological response) or event (RFS: first occurrence of recurrence or death from any cause; OS: death from any cause). Pathological response rates were measured in patients with a pathological response assessment. RFS rates were measured only in patients who received surgery (see Extended Data Table 1 for reasons for surgical omission). OS rates were measured in the whole cohort. Four patients were treated with CTLA-4 alone, hence PD-1 and PD-1 + IO groups may not sum to ICIs. Indented characteristics or disposition factors are subsets of the preceding.

^a^PD-1 includes both anti-PD-1 and anti-PD-L1 agents.

^b^IO includes any immuno-oncology agent(s), including other ICIs.

NR, not reached.

Within the ICI cohort, patients treated with PD-1 + IO had higher rates of MPR (61.4% versus 49.5%) and lower rates of pNR (27.5% versus 37.0%) than those treated with PD-1 alone (Table 2). The MPR rate was 62.5% with PD-1 + CTLA-4, 63.2% with PD-1 + LAG3 and 50.0% with PD-1 + other IO (Extended Data Table 2). In multivariate models, MPR but not pCR was significantly associated with type of ICI treatment (PD-1 alone or PD-1 + IO; Extended Data Table 4 and Supplementary Table 4).

### Radiological and metabolic response

Imaging responses per CT and fluorodeoxyglucose positron emission tomography (FDG-PET) scans are available in Supplementary Results 1, Extended Data Table 5 and Supplementary Fig. 1.

### Event-free survival

**Fig. 1:**
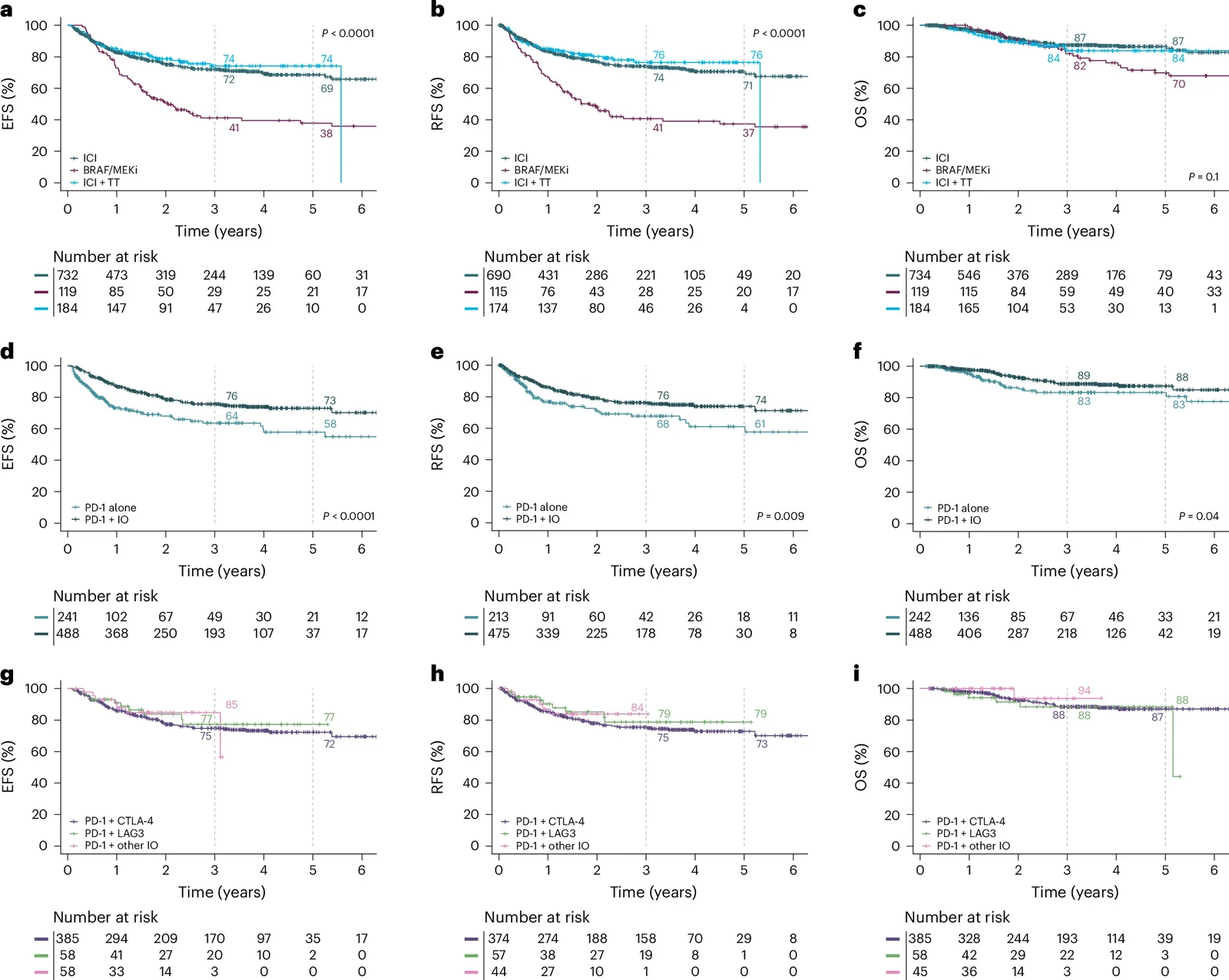
Survival outcomes by neoadjuvant regimen. **a**–**i**, EFS, RFS and OS rates by neoadjuvant regimen: ICIs versus BRAF/MEKis versus ICI + TT; PD-1 alone versus PD-1 + IO; and PD-1 + CTLA-4, PD-1 + LAG3 and PD-1 + other IO. Data labels are rounded to the nearest whole percentage. The log(rank *P*) < 0.0001 is specified below. **a**–**c**, ICIs versus BRAF/MEKis versus ICI + TT. **a**, At 3 years, the EFS rates were 72.0% (95% CI 68.3–75.9%) with ICIs, 41.2% (32.6–52.0%) with BRAF/MEKis and 74.2% (67.2–81.8%) with ICI + TT; at 5 years, EFS rates were 68.5% (95% CI 64.3–73.0%), 37.9% (29.2–49.1%) and 74.2% (67.2–81.8%), respectively. The exact log(rank *P*) < 6 × 10^−9^. **b**, At 3 years, the RFS rates were 74.0% (95% CI 70.2–77.9%) with ICIs, 40.7% (32.1–51.5%) with BRAF/MEKis and 76.4% (69.4–84.1%) with ICI + TT; at 5 years, RFS rates were 70.6% (95% CI 66.3–75.2%), 37.4% (28.7–48.7%) and 76.4% (69.4–84.1%), respectively. The exact log(rank *P*) < 8 × 10^−12^. **c**, At 3 years, the OS rates were 87.4% (95% CI 84.5–90.5%) with ICIs, 82.0% (74.4–90.3%) with BRAF/MEKis and 83.9% (77.4–90.9%) with ICI + TT; at 5 years, OS rates were 86.5% (95% CI 83.3–89.8%), 69.8% (60.2–80.9%) and 83.9% (77.4–90.9%), respectively. **d**–**f**, PD-1 alone versus PD-1 + IO. **d**, At 3 years, the EFS rates were 63.6% (95% CI 56.2–71.9%) with PD-1 alone and 75.7% (71.5–80.1%) with PD-1 + IO; at 5 years, EFS rates were 57.8% (95% CI 49.0–68.1%) and 73.0% (68.4–78.0%), respectively. The exact log(rank *P*) < 3 × 10^−5^. **e**, At 3 years, the RFS rates were 67.7% (95% CI 59.8–76.7%) with PD-1 alone and 76.3% (72.1–80.7%) with PD-1 + IO; at 5 years, RFS rates were 61.0% (95% CI 51.4–72.4%) and 73.9% (69.3–78.9%), respectively. **f**, At 3 years, the OS rates were 83.3% (95% CI 76.9–90.2%) with PD-1 alone and 88.7% (85.4–92.2%) with PD-1 + IO; at 5 years, OS rates were 83.3% (95% CI 76.9–90.2%) and 87.5% (83.7–91.4%), respectively. **g**–**i**, PD-1 + CTLA-4, PD-1 + LAG3 and PD-1 + other IO. **g**, At 3 years, the EFS rates were 74.8% (95% CI 70.2–79.7%) with PD-1 + CTLA-4, 77.3% (65.3–91.4%) with PD-1 + LAG3 and 84.7% (74.1–96.9%) with PD-1 + other IO; at 5 years, EFS rates were 72.3% (95% CI 67.2–77.7%) with PD-1 + CTLA-4 and 77.3% (65.3–91.4%) with PD-1 + LAG3. The 5-year data were not available for PD-1 + other IO. **h**, At 3 years, the RFS rates were 75.4% (95% CI 70.8–80.4%) with PD-1 + CTLA-4, 78.7% (66.9–92.7%) with PD-1 + LAG3 and 83.8% (72.6–96.7%) with PD-1 + other IO; at 5 years, RFS rates were 72.8% (95% CI 67.7–78.4%) with PD-1 + CTLA-4 and 78.7% (66.9–92.7%) with PD-1 + LAG3. The 5-year data were not available for PD-1 + other IO. **i**, At 3 years, the OS rates were 88.4% (95% CI 84.7–92.3%) with PD-1 + CTLA-4, 88.4% (79.0–98.8%) with PD-1 + LAG3 and 93.8% (82.6–100%) with PD-1 + other IO; at 5 years, OS rates were 87.0% (95% CI 82.9–91.3%) with PD-1 + CTLA-4 and 88.4% (79.0–98.8%) with PD-1 + LAG3. The 5-year data were not available for PD-1 + other IO.

EFS was defined as the time from first dose of neoadjuvant therapy to the first occurrence of unresectable preoperative progression, recurrence or death from any cause and is shown in Fig. 1. Overall, 287 (27.6%) patients had an event. This included 32 patients with unresectable preoperative progression, the rate of which was 3.8% (*n* = 27) with ICIs overall (8.3% (*n* = 20) with PD-1 alone and 1.4% (*n* = 7) with PD-1 + IO), 0.8% (*n* = 1) with BRAF/MEKis and 2.2% (*n* = 4) with ICI + TT.

The 5-year EFS rate was 68.5% (95% CI 64.3–73.0%) with ICIs, 37.9% (95% CI 29.2–49.1%) with BRAF/MEKis and 74.2% (95% CI 67.2–81.8%) with ICI + TT (Fig. 1). Among ICI-treated patients, the 5-year EFS was 57.8% (95% CI 49.0–68.1%) with PD-1 alone and 73.0% (95% CI 68.4–78.0%) with PD-1 + IO. The latter included a 5-year EFS rate of 72.3% (95% CI 67.2–77.7%) with PD-1 + CTLA-4 and 77.3% (95% CI 65.3–91.4%) with PD-1 + LAG3. The 5-year data were not available for PD-1 + other IO.

### Recurrence-free survival

Recurrence-free survival (RFS) was defined as the time from surgery to the first occurrence of recurrence or death from any cause and is shown in Fig. 1, Table 2 and Extended Data Table 2. The 5-year RFS rate was 70.6% (95% CI 66.3–75.2%) with ICIs, 37.4% (95% CI 28.7–48.7%) with BRAF/MEKis and 76.4% (95% CI 69.4–84.1%) with ICI + TT. With PD-1 alone, the 5-year RFS rate was 61.0% (95% CI 51.4–72.4%). With PD-1 + IO, the 5-year RFS rate was 73.9% (95% CI 69.3–78.9%). Within the PD-1 + IO cohort, the 5-year RFS rate was 72.8% (95% CI 67.7–78.4%) with PD-1 + CTLA-4 and 78.7% (66.9–92.7%) with PD-1 + LAG3; the 5-year data were not available for PD-1 + other IO.

Patients with MPR had 5-year RFS rates of 91.2% (95% CI 87.4–95.1%) with ICIs, 57.4% (95% CI 45.0–73.3%) with BRAF/MEKis and 89.6% (95% CI 81.5–98.5%) with ICI + TT, each higher than the nonstratified estimate for each regimen (Table 2). In ICI-treated patients with MPR, the 5-year RFS rate was 79.6% (95% CI 61.3–100%) with PD-1 alone and 92.8% (89.7–96.1%) with PD-1 + IO; the rates per PD-1 + IO subgroups are shown in Extended Data Table 2. Supplementary Fig. 2 visualizes how the MPR rate and 3-year RFS rate with MPR independently contribute to the RFS and OS benefits of neoadjuvant regimens. An interaction test between neoadjuvant treatment (PD-1 alone, PD-1 + IO, BRAF/MEKis and ICI + TT) and MPR was performed that was significant for RFS (*P* = 0.0113).

**Fig. 2:**
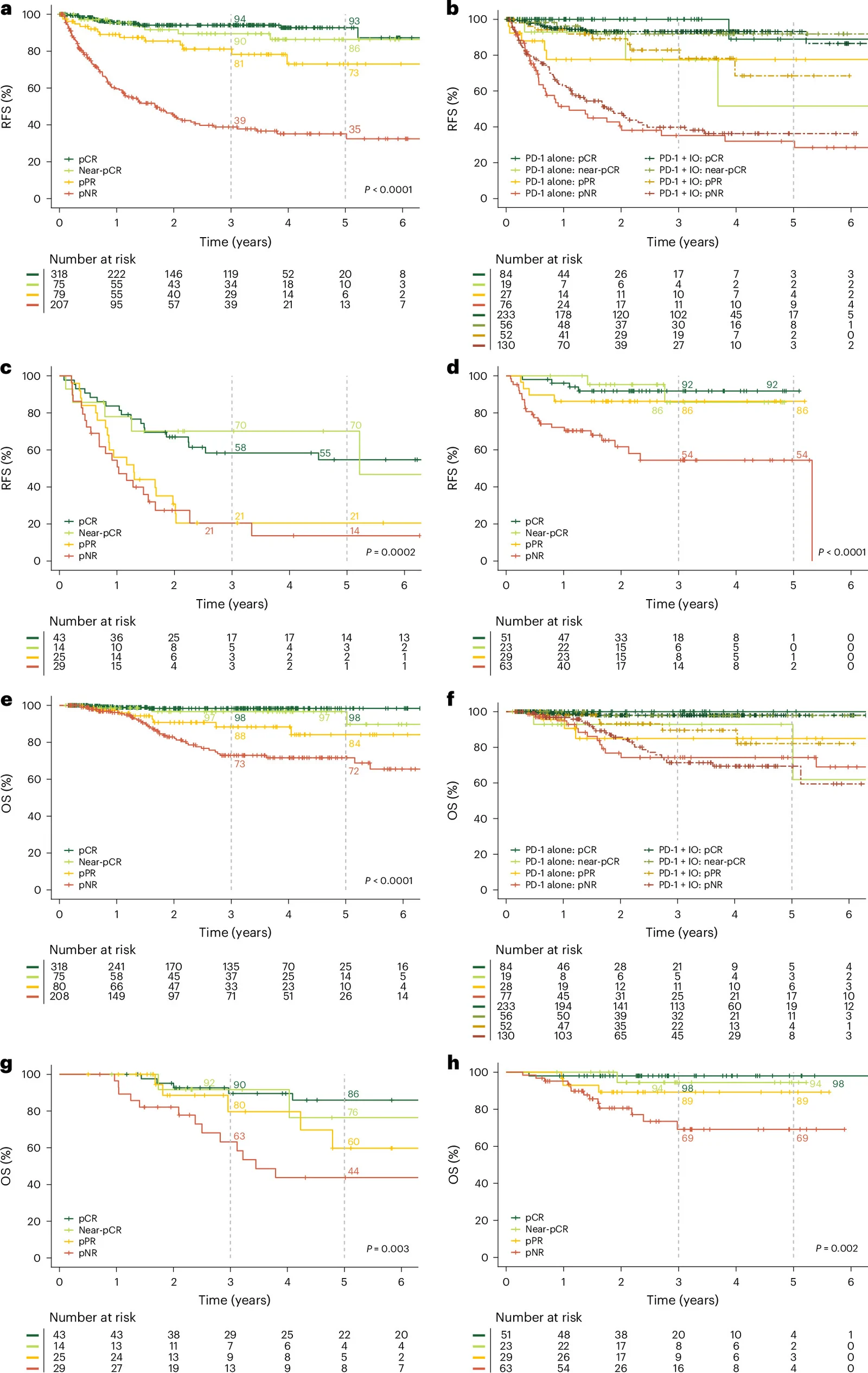
Survival outcomes by pathological response. **a**–**h**, RFS and OS shown by pathological response with ICIs, PD-1 alone and PD-1 + IO, BRAF/MEKis, and ICI + TT. Data labels are rounded to the nearest whole percentage. Rates with MPR are shown in Table 2. The log(rank *P*) < 0.0001 is specified below. **a**, RFS for ICIs. At 3 years, the RFS rates were 94.1% (95% CI 91.3–97.1%) with pCR, 89.5% (81.8–97.9%) with near-pCR, 81.1% (71.8–91.7%) with pPR and 38.9% (31.7–47.6%) with pNR; at 5 years, RFS rates were 92.7% (95% CI 88.8–96.8%), 86.4% (77.1–96.8%), 73.0% (60.0–88.8%) and 35.2% (27.8–44.4%), respectively. The exact log(rank *P*) = 2 × 10^−16^. **b**, PD-1 alone and PD-1 + IO. With PD-1 alone, the 3-year RFS rates were 100% with pCR, 77.4% (95% CI 52.6–100%) with near-pCR, 77.6% (61.8–97.3%) with pPR and 35.2% (23.9–51.8%) with pNR; at 5 years, the rates were 88.9% (95% CI 70.6–100%), 51.6% (21.2–100%), 77.6% (61.8–97.3%) and 32.0% (20.8–49.1), respectively. With PD-1 + IO, the 3-year RFS rates were 93.2% (95% CI 89.7–96.7%) with pCR, 91.8% (84.4–99.9%) with near-pCR, 82.9% (71.8–95.7%) with pPR and 39.8% (31.1–50.8%) with pNR; at 5 years, the rates were 93.2% (95% CI 89.7–96.7%), 91.8% (84.4–99.9%), 68.5% (49.8–94.2%) and 36.3% (27.5–47.8%), respectively. **c**, BRAF/MEKis. At 3 years, the RFS rates were 58.3% (95% CI 44.8–75.9%) with pCR, 70.1% (49.4–99.6%) with near-pCR, 20.5% (9.0–46.8%) with pPR and 20.5% (8.8–47.8%) with pNR; at 5 years, the RFS rates were 54.7% (95% CI 40.8–73.3%), 70.1% (49.4–99.6%), 20.5% (9.0–46.8%) and 13.7% (4.3–43.8%), respectively. **d**, ICI + TT. At 3 years, the RFS rates were 91.8% (95% CI 84.3–99.8%) with pCR, 85.7% (68.3–100) with near-pCR, 86.2% (74.5–99.7%) with pPR and 54.4% (41.0–72.2%) with pNR; at 5 years, the RFS rates were 91.8% (95% CI 84.3–99.8%), 86.2% (74.5–99.7%) and 54.4% (41.0–72.2%) with pCR, pPR and pNR, respectively. The 5-year data were not available for near-pCR. The exact log(rank *P*) = 4 × 10^−5^. **e**, OS for ICIs. At 3 years, the OS rates were 98.4% (95% CI 96.8–100%) with pCR, 96.6% (92.1–100%) with near-pCR, 88.3% (80.3–97.1%) with pPR and 72.9% (65.5–81.1%) with pNR; at 5 years, the OS rates were 98.4% (95% CI 96.8–100%), 96.6% (92.1–100%), 84.1% (73.5–96.3%) and 71.6% (64.0–80.1%), respectively. The exact log(rank *P*) = 4 × 10^−12^. **f**, PD-1 alone and PD-1 + IO. With PD-1 alone, the 3-year OS rates were 100% with pCR, 92.9% (95% CI 80.3–100%) with near-pCR, 85.0% (70.5–100%) with pPR and 74.3% (62.6–88.2%) with pNR; at 5 years, the rates were 100%, 92.9% (95% CI 80.3–100%), 85.0% (70.5–100%) and 74.3% (62.6–88.2%), respectively. With PD-1 + IO, the 3-year OS rates were 98.0% (95% CI 96.0–100%) with pCR, 97.8% (93.6–100%) with near-pCR, 89.6% (80.1–100%) with pPR and 71.4% (62.1–82.1%) with pNR; at 5 years, the rates were 98.0% (95% CI 96.0–100%), 97.8% (93.6–100%), 82.1% (67.0–100%) and 69.4% (59.7–80.7%), respectively. **g**, BRAF/MEKis. At 3 years, the OS rates were 89.5% (95% CI 80.3–99.9%) with pCR, 91.7% (77.3–100%) with near-pCR, 79.7% (61.0–100%) with pPR and 63.2% (46.3–86.3%) with pNR. At 5 years, the OS rates were 86.0% (95% CI 75.1–98.4%), 76.4% (51.4–100%), 59.8% (36.9–96.7%) and 43.8% (27.2–70.5%), respectively. **h**, ICI + TT. At 3 years, the OS rates were 98.0% (95% CI 94.3–100%) with pCR, 94.4% (84.4–100%) with near-pCR, 89.3% (78.5–100%) with pPR and 69.1% (55.3–86.4%) with pNR; at 5 years, the OS rates were 98.0% (95% CI 94.3–100%), 94.4% (84.4–100%), 89.3% (78.5–100%) and 69.1% (55.3–86.4%), respectively.

**Extended Data Fig. 1:**
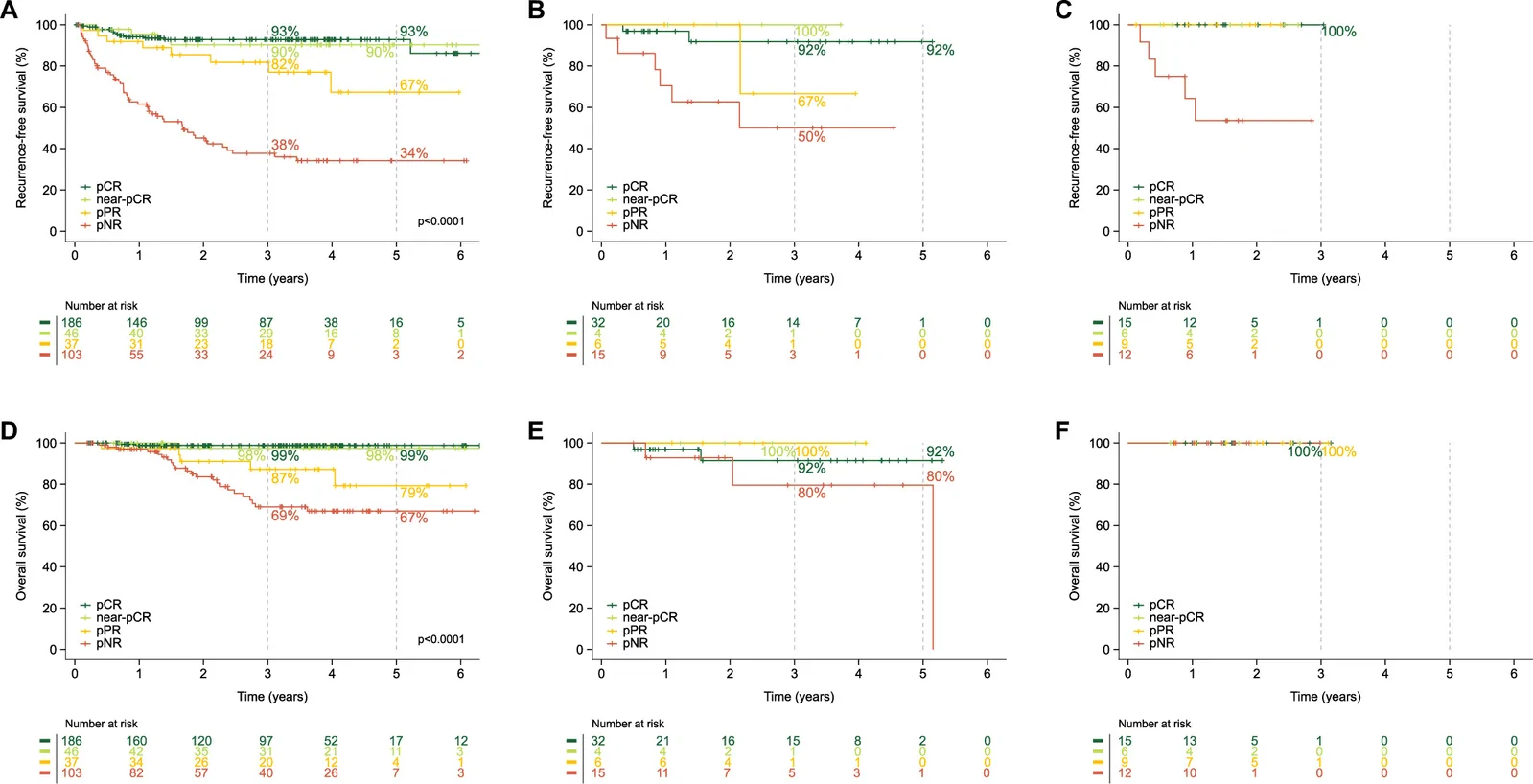
RFS and OS by pathological response in patients treated with PD-1 + CTLA-4, PD-1 + LAG3 or PD-1 + other IO (N = 489). **A–F**, RFS and OS shown by pathological response with PD-1 + IO combination therapies. Data labels are rounded to the nearest whole percentage. Rates with MPR are shown in Extended Data Table 2. The log(rank *P*) < 0.0001 is specified below. **A**,**D**, PD-1 + CTLA-4. **A**, At 3 years, RFS rates were 92.8% (95% CI 89.0–96.8%) with pCR, 90.3% (81.6–99.8%) with near-pCR, 81.8% (69.4–96.3%) with pPR and 37.7% (28.5–50.0%) with pNR. At 5 years, RFS rates were 92.8% (95% CI 89.0–96.8%), 90.3% (81.6–99.8%), 67.4% (48.4–93.8%) and 34.1% (24.9–46.7%), respectively. Exact log(rank *P*) < 2 × 10^−16^. **D**, At 3 years, OS rates were 98.8% (95% CI 97.2–100%) with pCR, 97.4% (92.4–100%) with near-pCR, 87.2% (76.2–99.9%) with pPR and 69.0% (58.9–81.0%) with pNR. At 5 years, OS rates were 98.8% (95% CI 97.2–100%), 97.4% (92.4–100%), 79.3% (62.9–99.9%) and 66.9% (56.4–79.4%), respectively. Exact log(rank *P*) < 4 × 10^−10^. **B**,**E**, PD-1 + LAG3. **B**, At 3 years, RFS rates were 91.8% (95% CI 81.2–100%) with pCR, 100% with near-pCR, 66.7% (30.0–100%) with pPR and 50.1% (27.4–91.8%) with pNR. At 5 years, the RFS rate was 91.8% (81.2–100%) with pCR. 5-year data was not available for near-pCR, pPR, or pNR. **E**, At 3 years, OS rates were 91.5% (95% CI 80.5–100%) with pCR, 100% with near-pCR, 100% with pPR and 79.6% (56.9–100%) with pNR. At 5 years, the OS rate was 91.5% (95% CI 80.5–100%) with pCR and 79.6% (56.9–100%) with pNR; 5-year data was not available for near-pCR or pPR. **C**,**F**, PD-1 + other IO. **C**, At 3 years, the RFS rate was 100% with pCR. No data was available for pCR at 5 years or near-pCR, pPR, or pNR at either landmark. **F**, At 3 years, the OS rate was 100% with pCR and 100% with pPR. No data was available for pCR or pPR at 5 years or near-pCR or pNR at either landmark. ICIs, immune checkpoint inhibitors; IO, immuno-oncology agent(s); MPR, major pathological response; NA, not applicable; OS, overall survival; pCR, complete pathological response; pNR, pathological non-response; pPR, pathological partial response; RFS, recurrence-free survival.

RFS by pathological response is shown in Fig. 2, Table 2 and Extended Data Table 2. In ICI-treated patients, pCR was associated with a 5-year RFS rate of 92.7% (95% CI 88.8–96.8%) and pNR with a 5-year RFS rate of 35.2% (95% CI 27.8–44.4%); these were similar among those who received PD-1 alone versus PD-1 + IO. Rates by pathological response to BRAF/MEKis and ICI + TT are also shown in Fig. 2 and by specific PD-1 + IO regimens in Extended Data Fig. 1.

RFS per radiological and metabolic response are shown in Supplementary Fig. 1. An alluvial plot of imaging responses and recurrence is shown in Supplementary Fig. 3.

### Overall survival

The OS was defined as the time from the first dose of neoadjuvant therapy to death from any cause and is shown in Fig. 1, Table 2 and Extended Data Table 2. At last follow-up, 117 (11.3%) patients had died, including 105 (10.1%) from melanoma, 1 (0.1%) due to treatment (previously reported^26,27^) and 11 (1.1%) from other or unknown causes. Of the 32 patients with unresectable preoperative progression, 12 (36.4%) had died, including 10 (37.0%) of 27 patients treated with ICIs; all deaths after unresectable preoperative progression occurred within 3 years of starting neoadjuvant therapy.

The 5-year OS rates were 86.5% (95% CI 83.3–89.8%) with ICIs, 69.8% (60.2–80.9%) with BRAF/MEKis and 83.9% (77.4–90.9%) with ICI + TT (Fig. 1). Within the ICI cohort, the 5-year OS was 83.3% (95% CI 76.9–90.2%) with PD-1 alone and 87.5% (83.7–91.4%) with PD-1 + IO. Within the PD-1 + IO cohort, the 5-year OS rates were 87.0% (95% CI 82.9–91.3%) with PD-1 + CTLA-4 and 88.4% (79.0–98.8%) with PD-1 + LAG3; the 5-year data were not available for PD-1 + other IO.

With MPR, the 5-year OS rates were 98.0% (95% CI 96.4–99.6%) with ICIs, 98.8% (96.4–100%) with PD-1 alone, 97.9% (96.1–99.8%) with PD-1 + IO, 84.2% (95% CI 73.8–96.0%) with BRAF/MEKis and 96.9% (95% CI 92.7–100%) with ICI + TT (Table 2). The rates per specific PD-1 + IO regimens are shown in Extended Data Table 2. An interaction test between neoadjuvant treatment (PD-1 alone, PD-1 + IO, BRAF/MEKis and ICI + TT) and MPR was performed that was not significant for OS (*P* = 0.3800).

In ICI-treated patients, the 5-year OS rate was 98.4% (95% CI 96.8–100%) after pCR and 71.6% (64.0–80.1%) after pNR (Fig. 2). The 5-year OS rate was equally excellent among patients achieving pCR after PD-1 alone (100%) or PD-1 + IO (98.0%, 95% CI 96.0–100%). The 5-year OS rates by pathological response to BRAF/MEKis and ICI + TT are also shown in Fig. 2.

### Adjuvant therapy

**Fig. 3:**
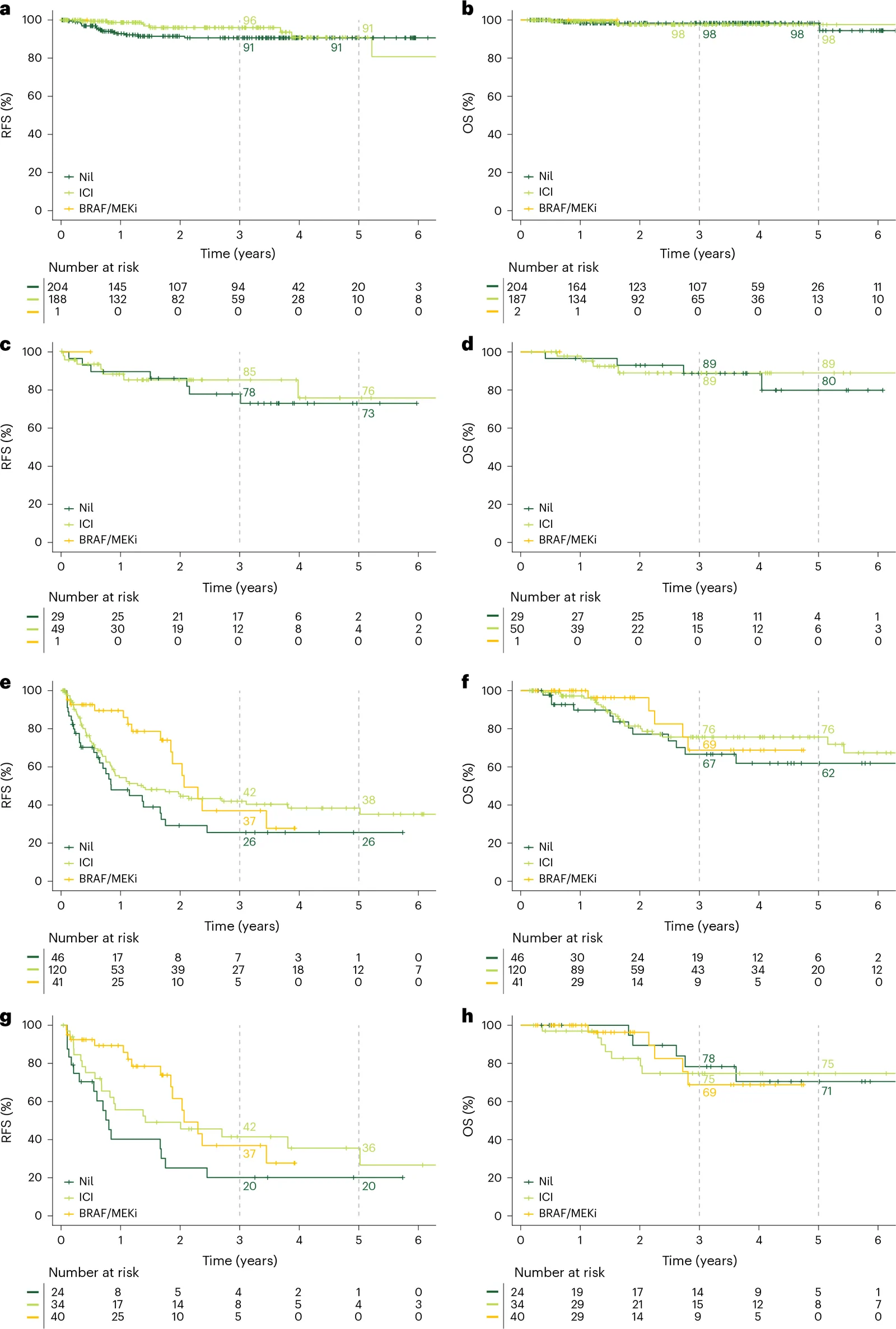
Survival outcomes with adjuvant systemic therapy after neoadjuvant ICIs. **a**–**i**, RFS and OS by adjuvant systemic therapy (ICIs, BRAF/MEKis or nil) in patients with MPR, pPR or pNR to neoadjuvant ICI. Data labels are rounded to nearest whole percentage. **a**,**b**, ICI-treated patients with MPR. **a**, At 3 years, the RFS rates were 90.6% (95% CI 86.3–95.2%) with nil adjuvant and 96.0% (92.6–99.5%) with adjuvant ICIs. At 5 years, the RFS rates were 90.6% (95% CI 86.3–95.2%) and 90.8% (83.5–98.9%) with nil and ICIs, respectively. Insufficient MPR patients received adjuvant BRAF/MEKis. **b**, At 3 years, the OS rates were 98.4% (95% CI 96.5–100%) with nil and 97.6% (94.9–100%) with ICIs; at 5 years, the OS rates were 98.4% (95% CI 96.5–100%) and 97.6% (94.9–100%) with nil and ICIs, respectively. Insufficient MPR patients received adjuvant BRAF/MEKis. **c**,**d**, ICI-treated patients with pPR. **c**, At 3 years, the RFS rates were 77.9% (95% CI 63.6–95.3%) with nil and 85.3% (75.0–97.1%) with ICIs; at 5 years, RFS rates were 73.0% (95% CI 57.5–92.7%) and 75.8% (58.2–98.8%) with nil and ICIs, respectively. Insufficient pPR patients received adjuvant BRAF/MEKis. **d**, At 3 years, the OS rates were 88.7% (95% CI 77.5–100%) with nil and 88.9% (79.0–100%) with ICIs; at 5 years, OS rates were 79.9% (95% CI 62.4–100%) and 88.9% (79.0–100%) with nil and ICIs, respectively. Insufficient pPR patients received adjuvant BRAF/MEKis. **e**,**f**, ICI-treated patients with pNR. **e**, At 3 years, the RFS rates were 25.6% (95% CI 14.3–45.8%) with nil, 42.0% (33.2–53.1%) with ICIs and 37.0% (20.2–67.9%) with BRAF/MEKi%; at 5 years, RFS rates were 25.6% (95% CI 14.3–45.8%) and 38.3% (29.3–50.1%) with nil and ICIs, respectively, The 5-year data were not available for BRAF/MEKis. **f**, At 3 years, the OS rates were 66.6% (95% CI 52.0–85.3%) with nil, 75.6% (66.7–85.7%) with ICIs and 68.8% (49.0–96.6%) with BRAF/MEKis; at 5 years, OS rates were 61.9% (95% CI 46.5–82.4) and 75.6% (66.7–85.7) with nil and ICIs, respectively. The 5-year data were not available for BRAF/MEKis. **g**,**h**, ICI-treated *BRAF* V600-mutant patients with pNR. **g**, At 3 years, the RFS rates were 20.1% (95% CI 8.4–47.9%) with nil, 41.5% (27.0–63.7%) with ICIs and 36.9% (20.1–67.7%) with BRAF/MEKis; at 5 years, RFS rates were 20.1% (95% CI 8.4–47.9%) and 35.5% (21.0–60.1%) with nil and ICIs, respectively. The 5-year data were not available for BRAF/MEKis. **h**, At 3 years, the OS rates were 78.3% (95% CI 61.5–99.6%) with nil, 74.7% (60.1–93.0%) with ICIs and 68.8% (49.0–96.6%) with BRAF/MEKis; at 5 years, OS rates were 70.5% (95% CI 51.3–96.8%) and 74.7% (60.1–93.0%) with nil and ICIs, respectively. The 5-year data were not available for BRAF/MEKis.

The impact of adjuvant systemic therapy was assessed in 681 ICI-treated patients with a pathological response assessment (Fig. 3). Of these, 358 (52.6%) received adjuvant ICI (PD-1 alone, *n* = 309; PD-1 + IO, *n* = 49), 44 (6.5%) received adjuvant BRAF/MEKis and 279 (41.0%) received no adjuvant systemic therapy. Among 46 patients with pNR who received no adjuvant therapy, 9 (19.6%) had ceased neoadjuvant treatment early due to toxicity (*n* = 8) or progression (*n* = 1).

Among ICI-treated patients with MPR, those who received no adjuvant drug therapy had similar RFS rates to those who received adjuvant ICIs (5-year RFS rates of 90.6% and 90.8%, respectively); 5-year OS rates were also similar among these groups. Adjuvant ICIs also had no RFS or OS impact on those with pPR. There were too few patients to assess the impact of adjuvant BRAF/MEKis in neoadjuvant ICI responders (MPR, *n* = 2; pPR, *n* = 1). In those with pNR, adjuvant BRAF/MEKis and adjuvant ICIs trended toward improved RFS rates (in both the overall and the *BRAF* V600-mutant pNR populations) and OS rates (in the overall pNR population).

### Clinical and molecular subgroups in patients treated with ICIs

#### *BRAF*-mutant melanomas

**Extended Data Fig. 2:**
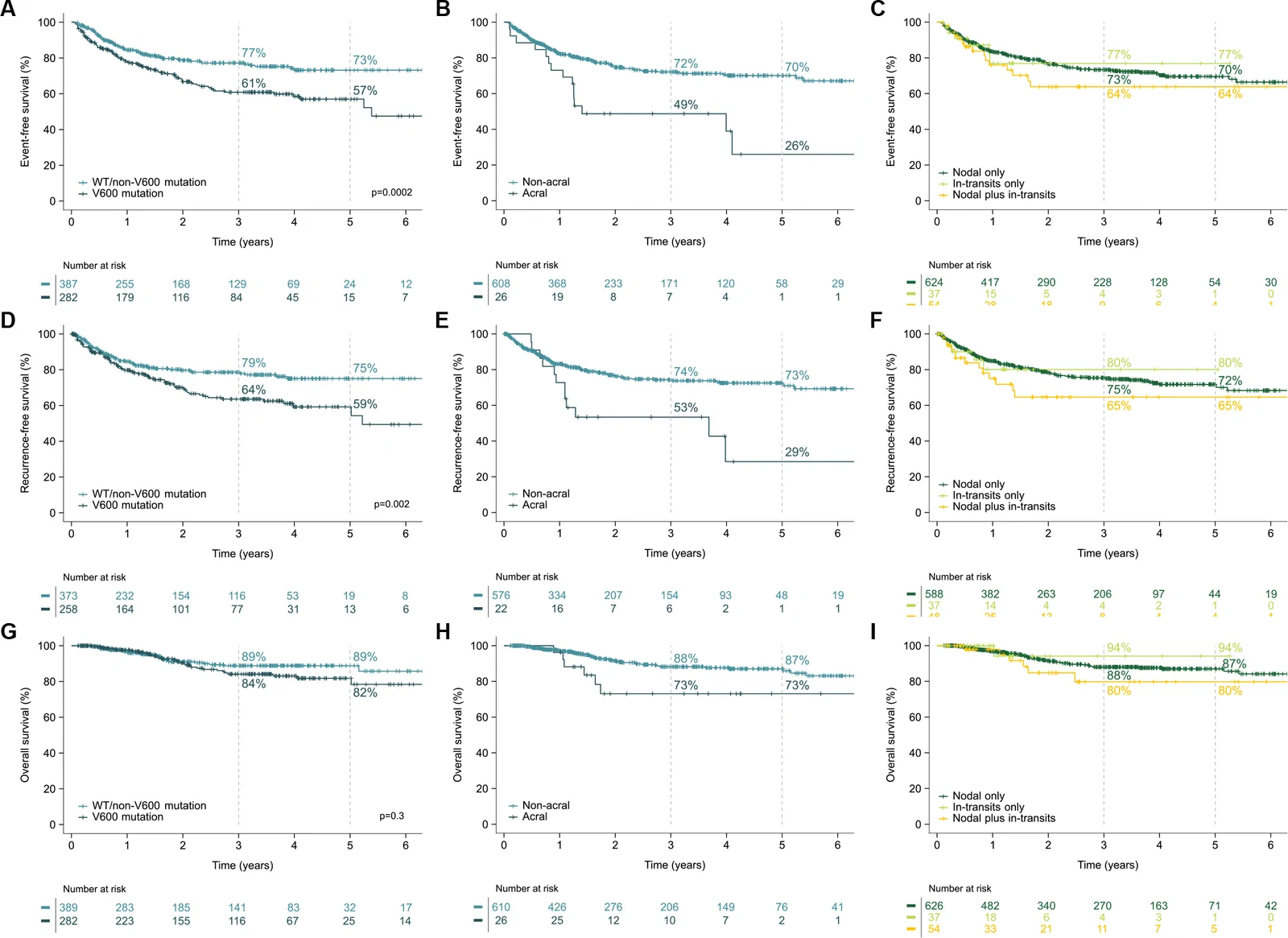
EFS, RFS and OS in patients with *BRAF* V600 mutation, acral melanoma or in-transit metastases treated with ICIs (N = 735). **A**–**I**, EFS, RFS and OS rates in ICI-treated patients with *BRAF* V600 mutation, acral melanoma or in-transit metastases. Data labels are rounded to the nearest whole percentage. **A**,**D**,**G**, *BRAF* V600 mutation. **A**, At 3 years, EFS rates were 77.2% (95% CI 72.6–82.2%) with WT/non-V600 mutation and 60.9% (54.5–67.9%) with V600 mutation. At 5 years, EFS rates were 73.2% (95% CI 67.7–79.2%) and 57.0% (49.9–65.1%), respectively. **D**, At 3 years, RFS rates were 78.6% (95% CI 73.9–83.5%) with WT/non-V600 mutations and 63.6% (56.9–71.0%) with V600 mutation. At 5 years, RFS rates were 75.0% (95% CI 69.5–81.0%) and 59.2% (51.6–68.0%), respectively. **G**, At 3 years, OS rates were 88.8% (95% CI 85.0–92.9%) with WT/non-V600 mutations and 84.1% (78.9–89.6%) with V600 mutation. At 5 years, OS rates were 88.8% (95% CI 85.0–92.9%) and 81.8% (75.9–88.1%), respectively. **B**,**E**,**H**, Acral melanoma. **B**, At 3 years, EFS rates were 72.2% (95% CI 68.0–76.6%) with non-acral and 48.7% (32.5–72.9%) with acral. At 5 years, EFS rates were 70.1% (95% CI 65.6–74.9%) and 26.0% (9.6–70.4%), respectively. **E**, At 3 years, RFS rates were 74.3% (95% CI 70.1–78.8%) with non-acral and 53.4% (35.8–79.6%) with acral. At 5 years, RFS rates were 72.5% (95% CI 68.0–77.4%) and 28.5% (10.5–77.1%), respectively. **H**, At 3 years, OS rates were 88.2% (95% CI 84.8–91.7%) with non-acral and 73.1% (56.5–94.5%) with acral. At 5 years, OS rates were 87.0% (95% CI 83.4–90.8%) and 73.1% (56.5–94.5%), respectively. **C**,**F**,**I**, In-transit metastases. **C**, At 3 years, EFS rates were 73.4% (95% CI 69.5–77.4%) with nodal only, 76.9% (61.7–95.9%) with ITMs only and 63.8% (50.3–81.1%) with nodal plus ITMs. At 5 years, EFS rates were 69.6% (95% CI 65.1–74.3%), 76.9% (61.7–95.9%) and 63.8% (50.3–81.1), respectively. **F**, At 3 years, RFS rates were 75.4% (95% CI 71.5–79.6%) with nodal only, 80.1% (65.5–98.0%) with ITMs only and 64.6% (50.3–82.9%) with nodal plus ITMs. At 5 years, RFS rates were 71.7% (95% CI 67.2–76.6%), 80.1% (65.5–98.0%) and 64.6% (50.3–82.9%), respectively. **I**, At 3 years, OS rates were 88.0% (95% CI 85.0–91.2%) with nodal only, 94.1% (83.6–100%) with ITMs only and 79.8% (66.0–96.4%) with nodal plus ITMs. At 5 years, OS rates were 87.0% (95% CI 83.7–90.5%), 94.1% (83.6–100%) and 79.8% (66.0–96.4%), respectively. EFS, event-free survival; ICIs, immune checkpoint inhibitors; ITMs, in-transit metastases; OS, overall survival; RFS, recurrence-free survival; WT, wild-type.

Of ICI-treated patients, 283 (38.5%) had a *BRAF* V600 mutation, 363 (49.4%) were *BRAF* wild-type and 26 (3.5%) had a non-V600 mutation. ICI-treated patients with a V600 mutation had lower MPR rates (46.3%) than wild-type patients (55.6%) or patients with non-V600 mutations (53.8%); the pNR rates were 34.6%, 26.4% and 23.1%, respectively. *BRAF* V600-mutant patients treated with PD-1 + IO had higher MPR rates than those treated with PD-1 alone (51.0% versus 35.6%) and higher pCR rates (38.5% versus 26.7%, respectively). The pCR rate was 50.0% (13) in those with non-V600 mutations, including 4 of 6 patients (66.7%) treated with PD-1 alone and 9 of 20 patients (45.0%) treated with PD-1 + IO. On multivariate analysis, the *BRAF* V600 mutation was a poor predictive factor for pCR (Supplementary Table 4), but not MPR (Extended Data Table 4). EFS and RFS were both poorer in patients with a *BRAF* V600 mutation compared with other patients (*BRAF* wild-type and non-V600 patients combined), but there was no difference in OS (Extended Data Fig. 2).

#### Acral melanomas

The ICI cohort included 26 patients with acral melanoma (Extended Data Table 6). The rate of unresectable preoperative progression was 11.5% (PD-1 alone, *n* = 2; PD-1 + CTLA-4, *n* = 1). Of 21 patients with pathological assessment, 8 had MPR (38.1%; pCR, *n* = 4; near-pCR, *n* = 4), 1 (4.8%) had pPR and 12 (57.1%) had pNR. At the last follow-up, 12 (46.2%) were alive and recurrence free, 8 (30.8%) were living with recurrence and 6 (23.1%) had died. Of the six deceased, five (83.3%) were treated with PD-1 alone and all had either pNR (*n* = 4) or unresectable preoperative progression (*n* = 2). Three of the four patients with pNR and death had received adjuvant ICI. At 5 years, patients with acral melanoma had an EFS rate of 26.0% (95% CI 9.6–70.4%), an RFS rate of 28.5% (95% CI 10.5–77.1%) and an OS rate of 73.1% (95% CI 56.5–94.5%) (Extended Data Fig. 2). All deaths occurred within 2 years of starting neoadjuvant therapy.

#### In-transit metastases

Of the 718 ICI-treated patients with stage III melanoma, 87.2% (626) had nodal disease alone, 5.3% (38) had in-transit (including satellite) metastases alone and 7.5% (54) had both nodal and in-transits (including satellites). The MPR rate was 58.2% (339) with nodal alone, 66.7% (24) with in-transits alone and 51.1% (24) with concurrent nodal and in-transit metastases. The 5-year EFS rate was 69.6% (95% CI 65.1–74.3) with nodal disease alone, 76.9% (61.7–95.9) with in-transit metastases alone and 63.8% (50.3–81.1) with both nodal disease and in-transits; similar trends were found in RFS and OS (Extended Data Fig. 2).

#### Oligometastases

Seventeen (2.3%) patients in the ICI cohort had stage IV disease, of whom six (35.3%) had pCR, one (5.9%) had near-pCR, two (11.8%) had pPR and eight (47.1%) had pNR (Extended Data Table 7). At a median follow-up of 2.1 (95% CI 1.4 to NR) years, 14 (82.4%) patients were alive and 3 (17.6%) patients had died. Of the three deaths, two occurred in patients with pNR and one in a patient with near-pCR. The patient with near-pCR had an isolated brain metastasis that was treated with neoadjuvant nivolumab 3 mg kg^−1^. The drug was stopped early due to toxicity and the lesion could not be definitively resected. Approximately 2 years after the first dose of neoadjuvant therapy, the patient recurred with multiple new lesions in the brain and lung, and death from melanoma occurred at 5 years. At last follow-up, five (83.3%) of the six patients with pCR were alive and recurrence free.

### Trial versus nontrial outcomes

**Extended Data Fig. 3:**
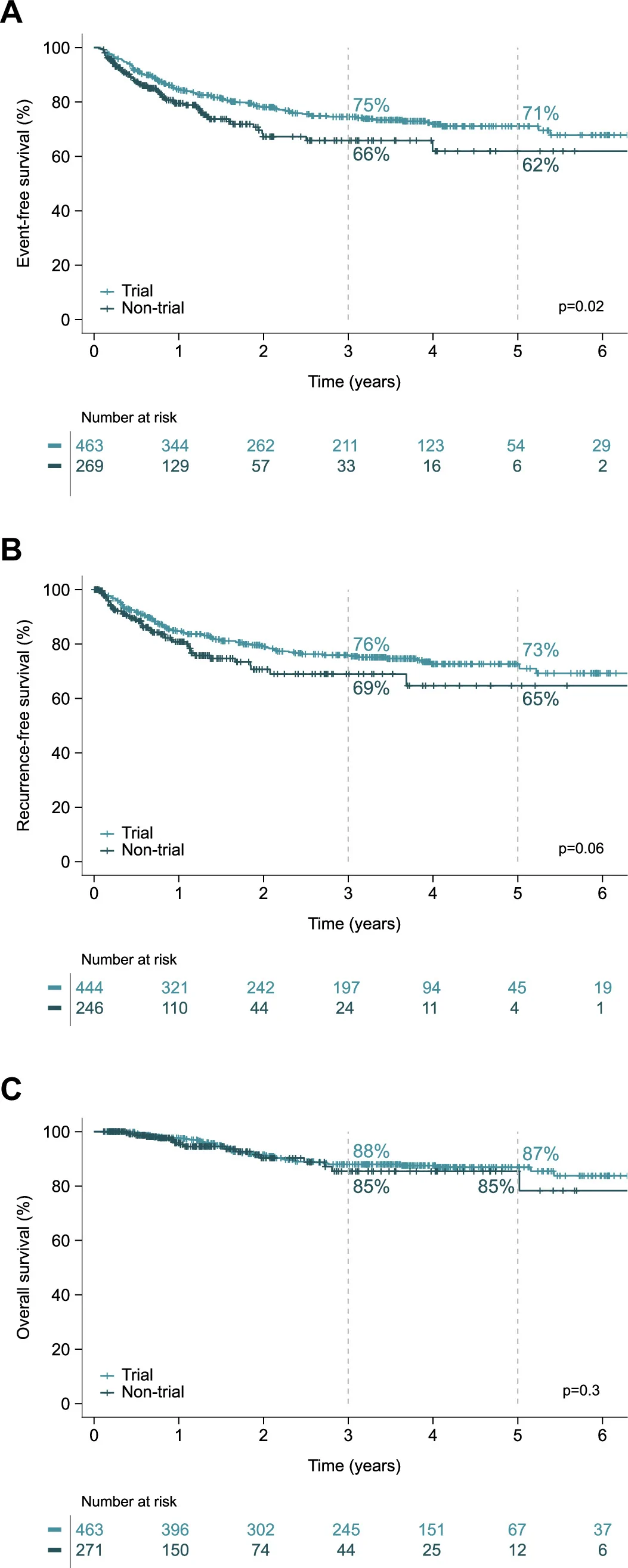
EFS, RFS and OS by treatment setting in patients treated with ICIs (N = 735). **A**–**C**, EFS, RFS and OS in trial versus nontrial patients treated with neoadjuvant ICIs. **A**, At 3 years, EFS rates were 74.5% (95% CI 70.4–79.0%) in trial patients and 65.8% (58.3–74.3%) in nontrial patients. At 5 years, EFS rates were 71.1% (95% CI 66.4–76.1%) and 61.9% (52.3–73.4%), respectively. **B**, At 3 years, RFS rates were 75.9% (95% CI 71.7–80.4%) in trial and 69.0% (61.1–77.9%) in nontrial. At 5 years, RFS rates were 72.6% (95% CI 67.9–77.8%) and 64.7% (54.3–77.1%), respectively. **C**, At 3 years, OS rates were 88.0% (95% CI 84.7–91.4%) in trial and 85.4% (78.4–93.0%) in nontrial. At 5 years, OS rates were 86.9% (95% CI 83.3–90.6%) and 85.4% (78.4–93.0%), respectively. EFS, event-free survival; ICIs, immune checkpoint inhibitors; OS, overall survival; RFS, recurrence-free survival.

Pathological response rates were similar among trial and nontrial patients, with pCR rates of 42.5% and 45.4%, respectively, and pNR rates of 31.7% and 30.1%, respectively; MPR rates were 58.9% and 55.6%, respectively. Nontrial patients had poorer EFS and RFS than patients treated within trial settings (Extended Data Fig. 3).

### Male versus female outcomes

There were no differences in EFS, RFS or OS between patients of male and female sex (Supplementary Fig. 4).

## Discussion

Neoadjuvant immunotherapy is moving rapidly from clinical trials into routine care^18–21^, but important knowledge gaps remain. We pooled participant-level data from 1,038 patients across 26 international sites. Neoadjuvant combination immunotherapy with anti-PD-1 plus any other IO elicited an MPR rate of 61.4%. Among patients with MPR to any ICI regimen, the 5-year OS rate was 98.0%, confirming that pathological response remains predictive across both single-agent and combination immunotherapy regimens.

Outcomes with neoadjuvant ICIs exceeded adjuvant benchmarks, with and without stratification by pathological response. ICI-treated patients had a 5-year RFS rate of 70.6%, comparing favorably with the ~50–55% 5-year RFS rates reported with adjuvant nivolumab (stage IIIB–IV)^29^, pembrolizumab (IIIA–D)^30^ and dabrafenib + trametinib (IIIA–D)^31^ (stage IIIA patients and early progressors are absent from neoadjuvant RFS analyses, although the latter are included in EFS).

The 5-year RFS rate with neoadjuvant BRAF/MEKis was 33 percentage points lower than with neoadjuvant ICI. There was unavoidable imbalance in the BRAF/MEKi cohort, namely a higher median SOD and enrichment for substage IIIC, reflecting the age of the NeoCombi trial, inclusion of stage IV patients and the high nodal disease burden associated with *BRAF* mutations. Despite this, our data clearly indicate that a neoadjuvant BRAF/MEKi is not effective. A neoadjuvant ICI regimen remains the best available treatment for *BRAF* V600-mutant patients, although pathological response rates are lower than in patients without V600 mutations. RFS with neoadjuvant BRAF/MEKis in our cohort was also inferior to that reported for adjuvant BRAF/MEKis in COMBI-AD^31^.

Whether neoadjuvant ICIs can be augmented with targeted therapies remains unclear. The ICI + TT cohort was heterogeneous, comprising seven different regimens in small numbers, which precludes firm conclusions. In the 60-patient NeoTrio study, adding BRAF/MEKis to anti-PD-1 in sequence or concurrently did not sufficiently alter survival to warrant use^23^. Multiclass drug trials should continue but focus on patients predicted to respond poorly to neoadjuvant ICIs^32^, providing a clean population to assess new agents, while protecting those who are likely already curable with current ICI regimens.

After neoadjuvant ICIs, histopathological assessment can identify which patients are treatment resistant and likely to recur or die from melanoma, allowing us to tease the ‘worst of the best’ (pNR) from the ‘best of the best’ (pCR). In patients who achieved MPR with combination immunotherapy, the 5-year RFS rate was 92.8%, 57 percentage points higher than with pNR. Survival outcomes appear equivalent among ICI-treated patients with near-pCR and pCR, supporting use of the MPR endpoint. Survival with near-pCR after anti-PD-1 + anti-CTLA-4 was 90.3% at 3 years, compared with 81.2% reported in NADINA^15^. The NADINA finding is under investigation and central review, but relates to only eight patients. Near-pCR rates are low (~7–15%) across neoadjuvant ICI studies^13,16,27,33,34^, which could increase variability in outcomes.

Neoadjuvant combination immunotherapy was more effective than anti-PD-1 alone, with 5-year RFS rates of 73.9% and 61.0%, respectively. CIs for 5-year RFS estimates with anti-PD-1 alone were wide and overlapping, partly reflecting shorter follow-up due to international approvals of neoadjuvant anti-PD-1 from late 2023. The difference in effectiveness is mainly driven by different rates of MPR (61.4% with anti-PD-1 + IO versus 49.5% with anti-PD-1 alone). Although 5-year RFS rates in patients with MPR had similarly wide CIs, 5-year OS estimates were narrower, approaching 100% in both cohorts. This suggests that the survival benefit with MPR is durable regardless of regimen, but longer follow-up is needed given the ceiling effect on the upper confidence bound. Data for immunotherapy combinations other than anti-PD-1 + anti-CTLA-4 remains limited. The 4-year EFS rate was 80% in a single-arm phase 2 trial of anti-PD-1 + anti-LAG3^35^. Greater cohort sizes and longer follow-up will help confirm the efficacy and application of newer anti-PD-1 + IO combinations^35–37^. The failure of combination immunotherapy in the adjuvant setting^38,39^ may reflect the impact of removing tumor-draining lymph nodes on T cell response to anti-CTLA-4 and anti-LAG3.

Our data suggest that patients with MPR or pPR after neoadjuvant ICIs gained no benefit from adjuvant ICIs; there was insufficient data to draw the same conclusion for adjuvant BRAF/MEKis. These results require prospective investigation to minimize bias by indication (we did not adjust for adjuvant therapy in primary survival analysis given this nonrandom assignment). A well-powered trial of adjuvant anti-PD-1 alone versus BRAF/MEKis in *BRAF*-mutant patients with pNR would be valuable. Overall, a substantial group of patients will not benefit from adjuvant therapy.

As neoadjuvant trials move toward de-escalation of nodal surgery^34^, the prognostic accuracy of less invasive measures, such as imaging responses, liquid biopsies and smaller resection specimens (for example, the largest or ‘index’ node^34,40^), needs to be established^41^. Pathological response was a better predictor of recurrence than CT or FDG-PET response and there is no conclusive evidence that omitting TLND based on imaging alone is appropriate. Patients with MPR in an index node who received no further surgery and no adjuvant therapy in the PRADO trial had excellent RFS outcomes^34,42^, suggesting that index node response is an effective prognostic indicator and that TLND and adjuvant drug may be net negative in neoadjuvant responders (the latter of which is supported by our data). Index node response has been shown to be representative of the total nodal basin^40^. Overall, insufficient evidence exists to omit TLND routinely. The randomized phase 3 MSLT-3 trial (NCT07049276) and phase 2 OMIT trial (NCT06754904) are addressing this question, although comparative analyses will become more challenging as more off-trial patients forgo surgery.

Predictive tools that integrate baseline multi-omic, radiological and clinical tests^43–45^ are becoming increasingly sophisticated. Whether these tools compare with the gold standard of pathological response needs prospective investigation. The ongoing NeoIRENIE trial (NCT06999980) uses one such model^45^ to predict response to approved neoadjuvant ICI regimens and allocate patients to standard or experimental immunotherapies. This allows ICI nonresponders to be identified upfront and randomized among intensified and nonstandard immunotherapy arms.

Given the effect size and rapid uptake of neoadjuvant immunotherapy, evidence and advice on the management of patients in clinical, histopathological and molecular subgroups (who have been excluded or poorly represented in trial cohorts) are critical.

Neoadjuvant ICIs were effective in patients with all presentations of stage IIIB–D melanoma, including those with in-transits alone, in-transits plus nodal disease or nodal disease alone. The ongoing NeoReNi II (NCT05418972) and MARIANE (NCT06240143) trials will determine whether neoadjuvant ICIs are useful in high-risk stage II melanoma. In our ICI-treated resectable stage IV cohort, 14 of 17 patients were alive and recurrence free at last follow-up and, although most received systemic adjuvant therapy, 3 recurrence-free patients did not. Alongside data in advanced melanoma^46^, this suggests that some stage IV patients can achieve long-term disease control with a short course of ICIs^47^.

Acral melanomas had a 38.1% MPR rate to neoadjuvant ICIs and survival outcomes were consistently worse than patients with non-acral melanomas. A retrospective study suggested that adjuvant anti-PD-1 alone may have a small benefit in stage IIIA–D acral melanoma compared with historical data (3-year RFS rate of 43% with adjuvant anti-PD-1 and 32% with surgery alone)^48^. In our small acral cohort, where stage ranged from IIIB–IV, the 3-year RFS rate was 53.4%. This suggests that neoadjuvant ICIs are likely a better option than adjuvant in the acral setting, although a dedicated prospective study is needed.

Retrospective cancer cohorts are prone to heterogeneity across stage, performance status, prior treatment, extent of visceral disease and length of survivorship, compounded by the biological selection pressure of prior systemic anticancer drugs. Strict requirements for neoadjuvant treatment in trials and per label, including that the patient has stage IIIB–D, systemic treatment-naive, clinically detected, resectable melanoma and is a surgical candidate, avoid or minimize many of these factors. Within our cohort, 96.6% of patients were stage III, 89.5% had nodal metastases and all were RECIST measurable, suggesting that most nontrial patients received treatment similar to clinical trials. Over 70% of patients in this cohort were treated in prospective INMC-informed trials, including almost all patients in the BRAF/MEKi (94.1%) and ICI + TT (97.8%) arms, which further reduced variation. Within the nontrial cohort, patients were treated at specialized cancer centers rather than in routine care. These factors limit the confounding that typically characterizes retrospective oncology datasets.

Limitations of the study include small subgroup sizes, particularly for in-transit metastases and acral melanoma, where dedicated prospective research is needed. Central review of histopathology or imaging was not feasible at this scale, which may have influenced response rates. Missing FDG-PET data reflect variation in its use across countries and sites.

Given the complexity of coordinating neoadjuvant treatment (which requires a multidisciplinary treating team and multifactorial decisions about managing early toxicities, extent of surgery and adjuvant therapy), nontrial data are of real interest. We found poorer EFS and RFS but similar OS in nontrial versus trial patients, although follow-up was shorter in the nontrial population. Again, the relative homogeneity of a neoadjuvant population would account for similar OS between trial and nontrial patients. High neoadjuvant expertise and established workflows across contributing centers may overestimate effectiveness in nontrial patients. Implementation of neoadjuvant therapy, particularly in nonspecialist cancer clinics without access to an established multidisciplinary treating team and linked pathology service, remains a critical responsibility of clinician–scientists (including the authors), professional societies, industry, government and other professionals.

This analysis provides a comprehensive overview of neoadjuvant therapy up to 2026. We confirm that pathological response is highly predictive of long-term survival in those treated with neoadjuvant single-agent or combination immunotherapy, stratifying patients into a favorable cohort (MPR) that can forgo adjuvant therapy and an unfavorable cohort (pNR) for whom personalized adjuvant strategies can be explored. Whether multiclass combinations can improve on pure ICIs is unclear; what is clear, however, is that neoadjuvant BRAF/MEKis can be largely abandoned. Neoadjuvant anti-PD-1-based immunotherapy is the preferred therapy for patients with resectable stage IIIB–D melanoma. Our findings support a framework for testing new adjuvant strategies in patients with pNR. Those with empirical (and, in future, predicted) resistance to current ICI regimens should be triaged to clinical trials.

## Methods

### Patients and treatment

This study comprised 1,038 patients with clinically detected, surgically resectable, AJCC 8th edn stage IIIB–IV cutaneous and acral melanoma, and included patients with nodal disease, in-transit (including satellite) disease and/or distant metastases. Patients were aged ≥18 years and of any sex (female, *n* = 397; male, *n* = 641; nonfemale or nonmale, *n* = 0) or gender. All patients received systemic neoadjuvant therapy with an ICI-based immunotherapy, BRAF/MEKis or ICI + any TT. Treatment regimens have been abbreviated earlier in tables, figures, extended data and Supplementary Information as per Supplementary Table 2. Neoadjuvant chemotherapy and intralesional therapy were excluded. Patients were included irrespective of whether they underwent surgery. Surgeries could include resection of in-transit or satellite disease, TLND, index node resection and/or metastasectomy, and could be followed by adjuvant therapy or not. There was no restriction on previous anticancer treatments. This study was endorsed by the Melanoma Institute Australia Research Committee and approved through the Royal Prince Alfred Hospital Ethics Review Committee (no. HREC/11/RPAH/32) under protocol nos. X15-0311 and 2019/ETH06854 (2019–2030). All sites obtained relevant local approvals and were provided with the Melanoma Institute Australia study protocol and data collection forms. Informed consent was obtained from all individual participants included in the study via the institutional general research registry at the time of admission. There was no participant compensation. All data were deidentified before analysis.

### Outcomes

Key outcomes included pathological response^28^, RECIST v1.1 response^49^, European Organisation for Research and Treatment of Cancer (EORTC) FDG-PET response^50^, EFS^51^, RFS and OS. Pathological response was only included if scored per the original 2018 INMC criteria^28^. Pathological response categories included pCR (defined as the complete absence of residual viable tumor in the treated tumor bed), near-pCR (defined as >0% and ≤10% of the tumor bed occupied by viable tumor cells), pPR (defined as >10% and ≤50% of the treated tumor bed occupied by viable tumor cells) and pNR (defined as >50% of the tumor bed occupied by viable tumor cells). MPR included those with pCR or near-pCR (that is, ≤10% residual viable tumor). Index node resection was considered a definitive resection only if pathological response was pCR. RECIST v1.1 response and EORTC FDG-PET response were calculated from CT scans and FDG-PET scans, respectively, at baseline and before surgery, in patients for whom data were available. Histopathology and imaging data were reviewed locally. EFS was defined as the time from the first dose of neoadjuvant therapy to the first occurrence of unresectable preoperative disease progression, recurrence or death from any cause. RFS was defined as the time from surgery to the first occurrence of recurrence or death from any cause. OS was defined as the time from first dose of neoadjuvant therapy to death from any cause.

### Statistical analysis

Patient-level data regarding demographics, baseline characteristics, treatment regimen, response to treatment (pathological, radiological and metabolic), recurrence, patterns and management of recurrence and survival were collected; patient sex was captured (from medical records), but not gender. Patient and disease characteristics were summarized using standard descriptive statistics: median (IQR) for continuous variables and frequency (proportion) for categorical variables. All response rates were provided along with corresponding two-sided 95% Clopper–Pearson exact CIs. Survival rates (EFS, RFS and OS) were analyzed using the Kaplan–Meier method and log(rank *P*) from the time of the first dose of neoadjuvant therapy, including landmark survival rates at 3 and 5 years and median survivals (with 95% CIs) when reached. Data were disaggregated for survival analysis by treatment, pathological response, extent of spread, mutation status, treatment setting and biological sex. Cox’s regression was used to generate predictive imaging models and area-under-the-curve values. A multivariate logistic regression analysis for MPR within the whole cohort was used to compare PD-1 alone with PD-1 + IO, BRAF/MEKis and ICI + TT. Potential confounders were selected from variables associated with MPR at a significance level of *P* ≤ 0.20 in univariate analyses and *P* values calculated using two-sided Wald’s tests. Variable selection for the multivariate models used to assess clinicopathological factors and MPR or pCR in patients treated with ICIs was performed using the penalized least absolute shrinkage and selection operator method and *P* values calculated using two-sided Wald’s tests. Interaction tests between MPR and neoadjuvant treatment type were assessed when comparing RFS and OS across treatment groups. Data were collected by investigators at each site and centrally collated and stored in Microsoft Excel for Microsoft 365 MSO (v2408 + 16.0.17928.20114) 64-bit. Statistical analyses were performed using R (v4.6.1 ‘Happy Hop’)^52^ with RStudio (v2025.06.0 + 242 ‘Blue Plumbago’ Release (d1dc28bb, 2026-06-18) for Windows 64-bit)^53^. R packages included: dplyr (v1.2.1)^54^, tidyr (v1.3.2)^55^, stringr (v1.6.0)^56^, forcats (v1.0.1)^57^, survival (v3.8-9)^58^, timeROC (v0.4.1)^59^, table1 (v1.5.1)^60^, ggplot2 (v4.0.3)^61^, survminer (v0.5.2)^62^, ggrepel (v0.9.8)^63^, ggtext (v0.1.2)^64^, ggalluvial (v0.12.6)^65^ and pacman (v0.5.1)^66^. Analyses were also performed using SAS (v9.4)^67^.

### Reporting summary

Further information on research design is available in the Nature Portfolio Reporting Summary linked to this article.

## Online content

Any methods, additional references, Nature Portfolio reporting summaries, source data, extended data, supplementary information, acknowledgements, peer review information; details of author contributions and competing interests; and statements of data and code availability are available at https://doi.org/10.1038/s41591-026-04677-z.

## Supplementary information

Supplementary Information Supplementary Results, Tables 1–4, Figs. 1–4 and References.

Reporting Summary

## Figures and Tables

**Extended Data Table 1:**
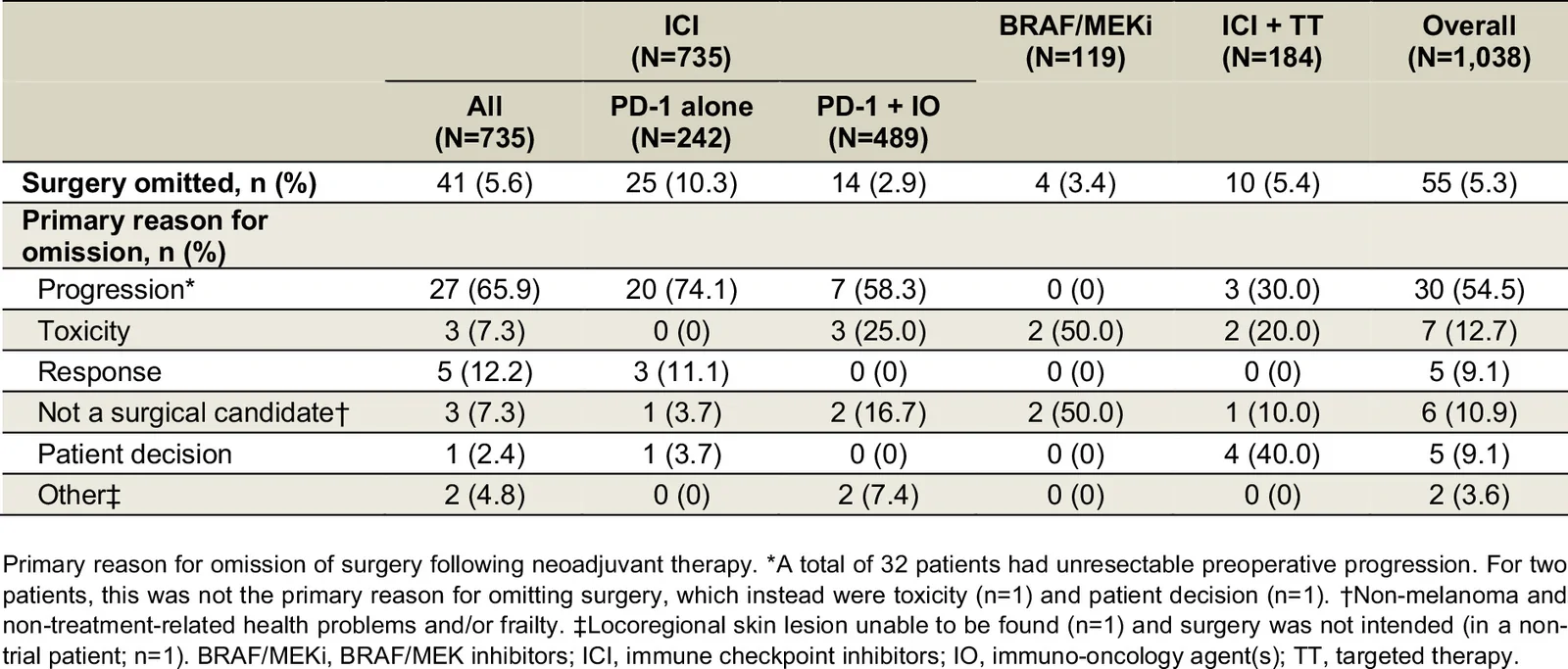
Reasons for omission of surgery in all patients (N = 1,038)

**Extended Data Table 2:**
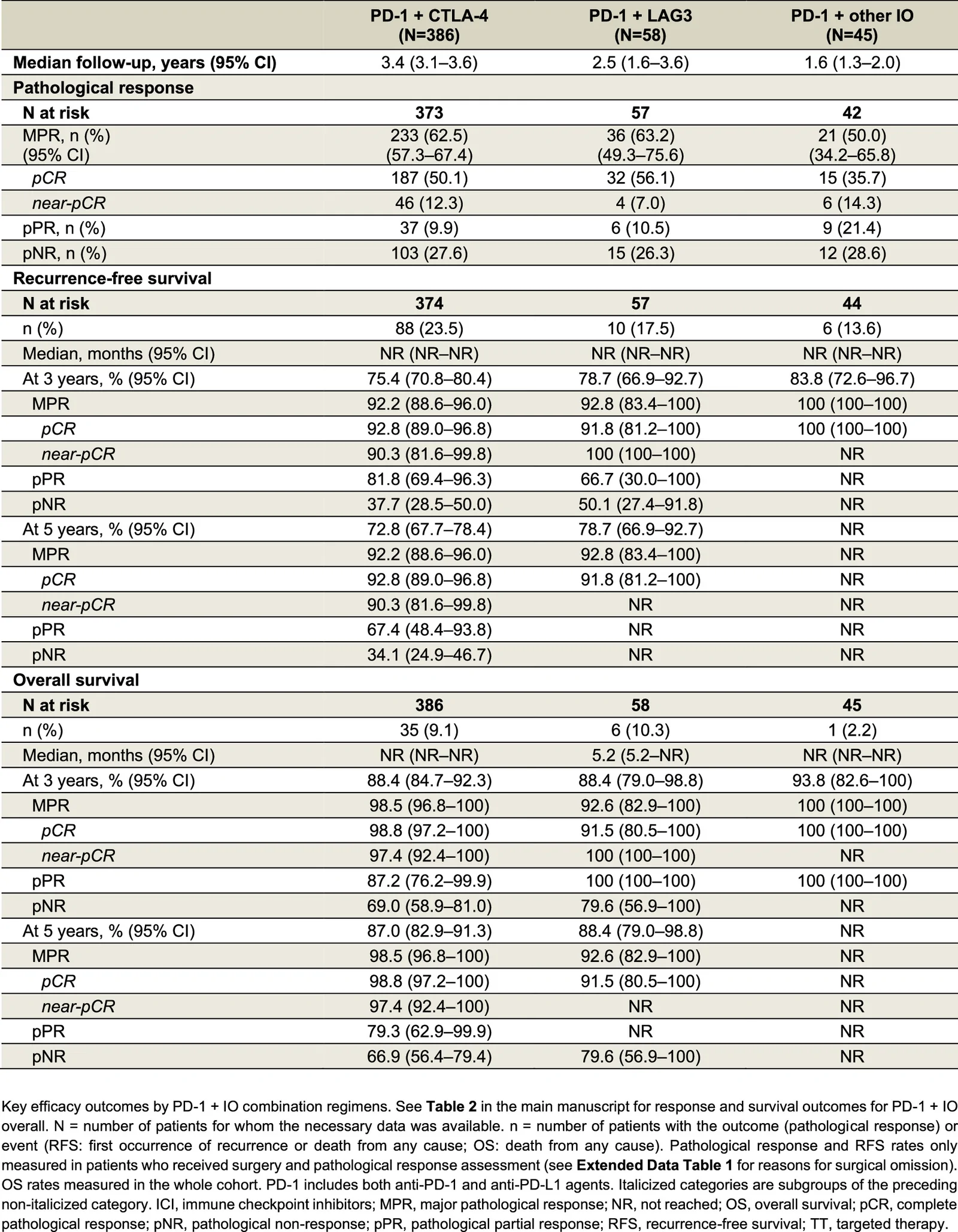
Pathological response and survival in patients treated with PD-1 + CTLA-4, PD-1 + LAG3, or PD-1 + other IO (N = 489)

**Extended Data Table 3:**
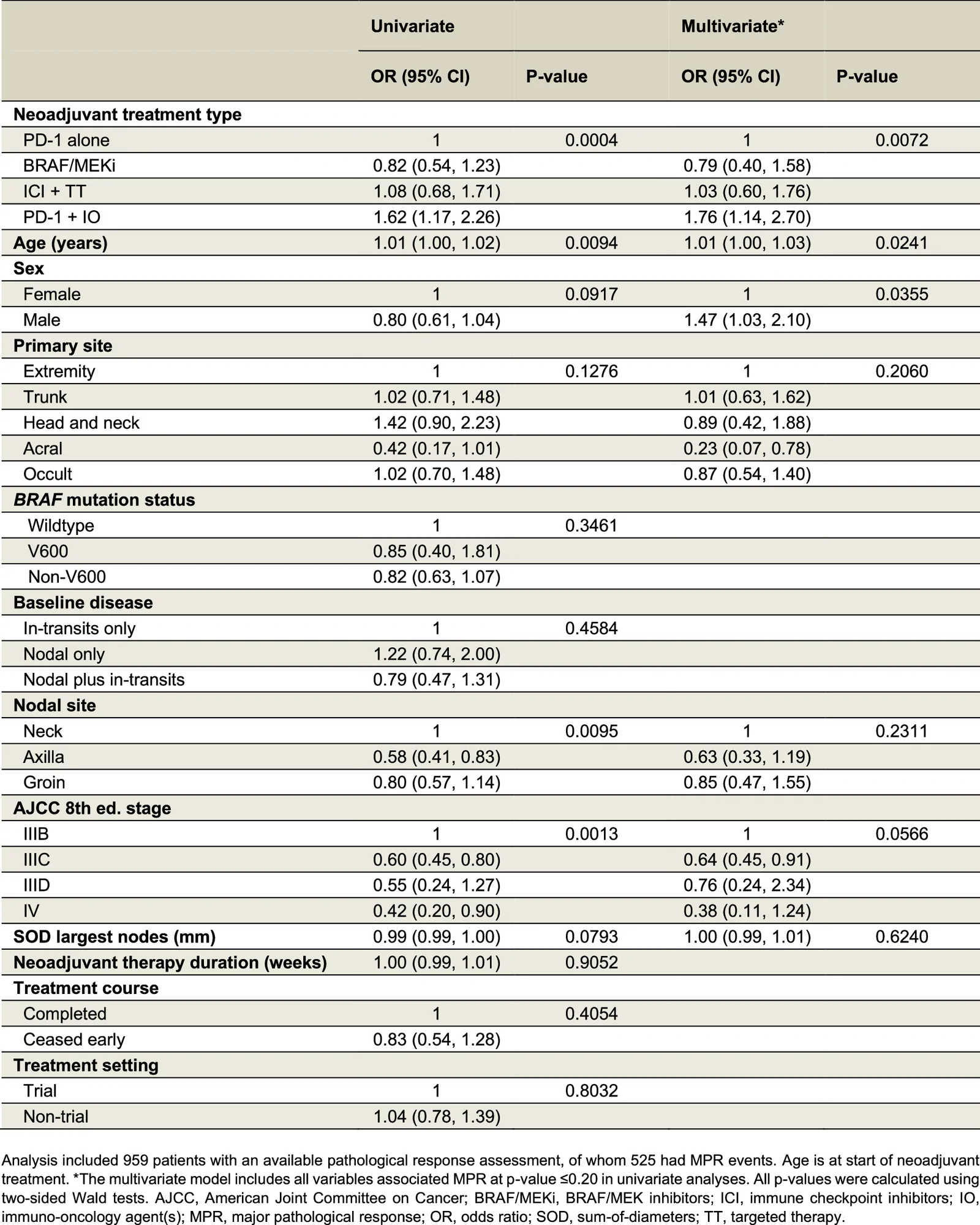
Effect of neoadjuvant treatment type on MPR after adjusting for potential clinicopathological confounders in all patients (N = 1,038)

**Extended Data Table 4:**
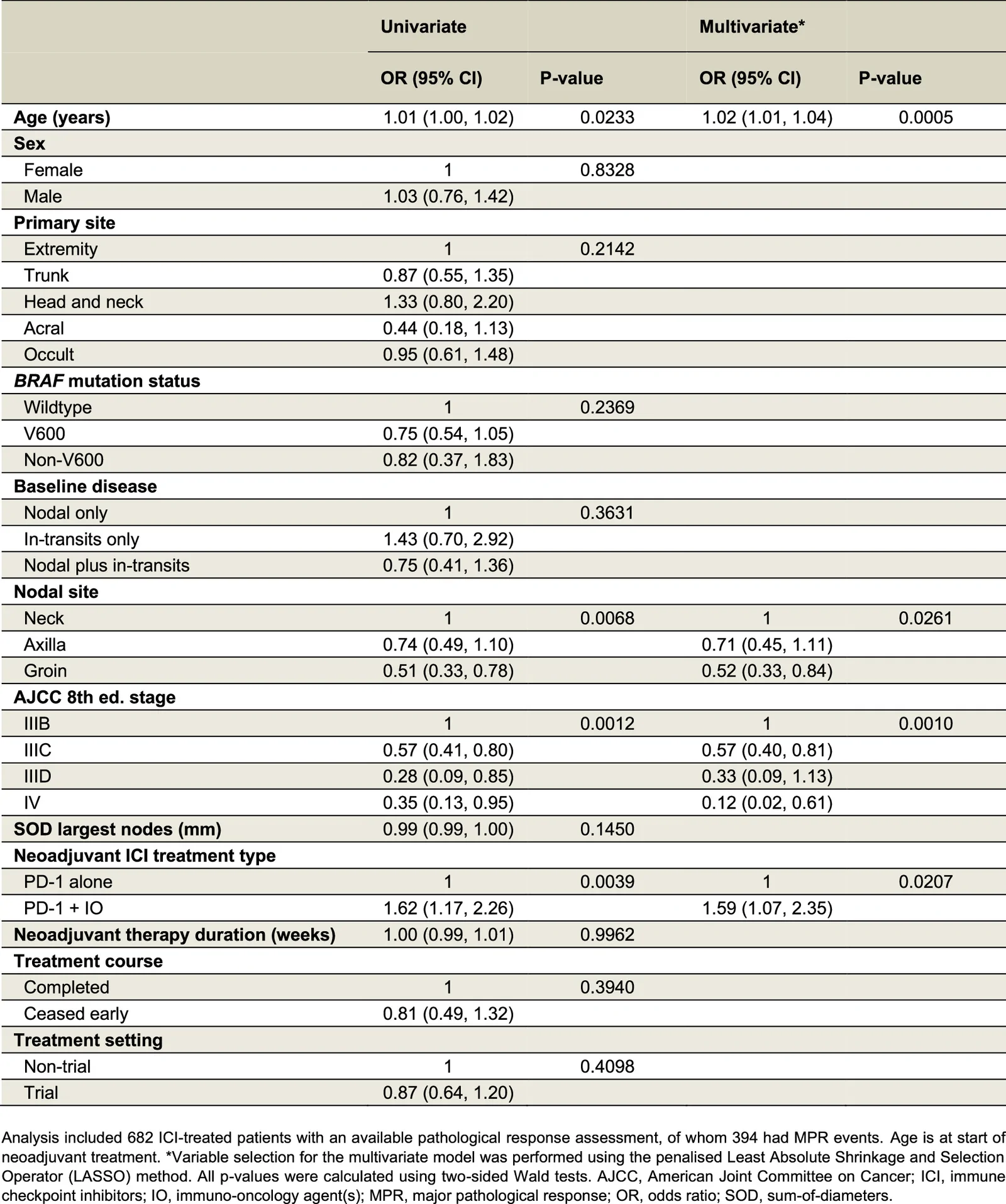
Clinicopathological factors associated with MPR in patients treated with ICI (N = 735)

**Extended Data Table 5:**
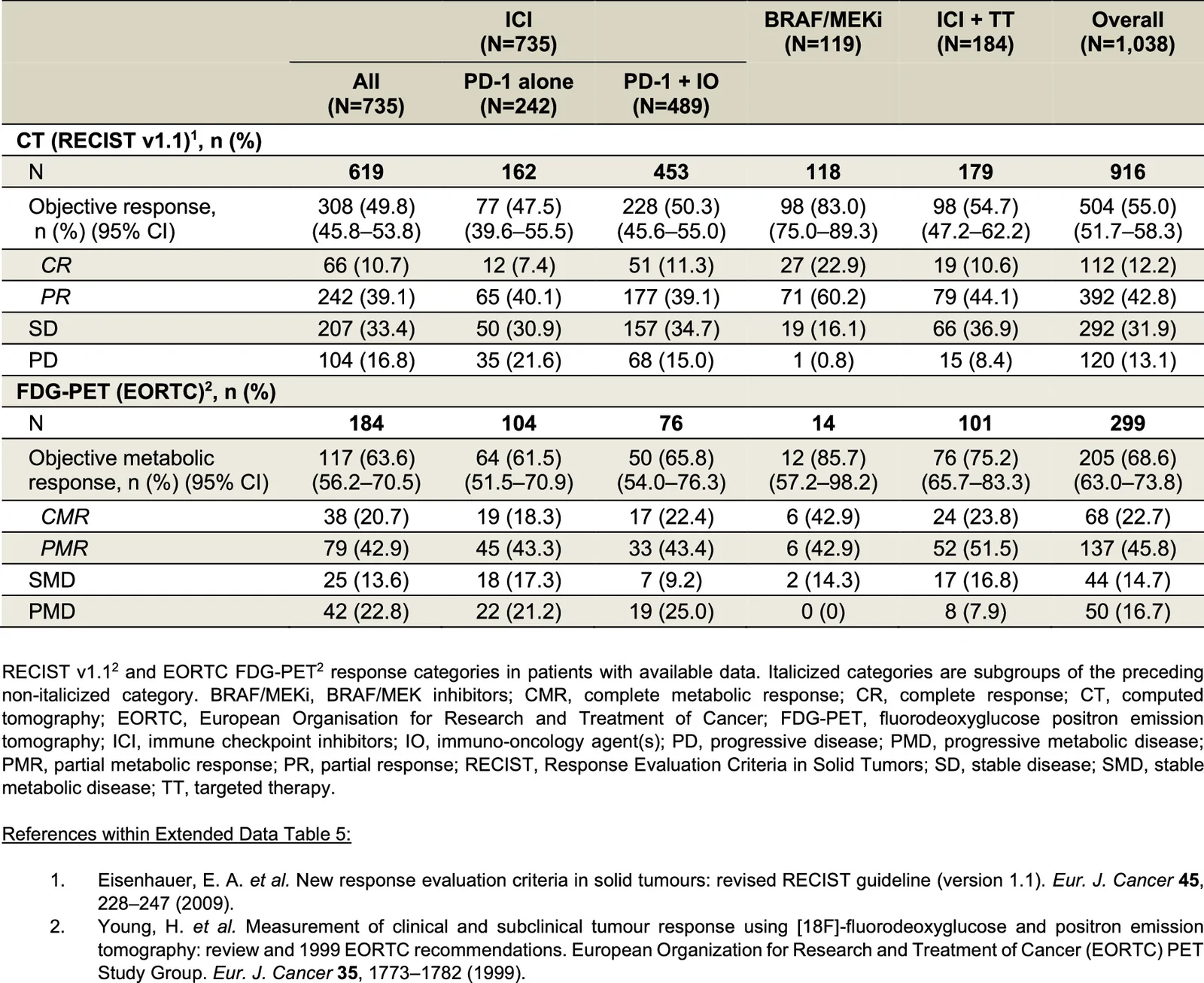
CT and FDG-PET response outcomes in all patients (N = 1,038)

**Extended Data Table 6:**
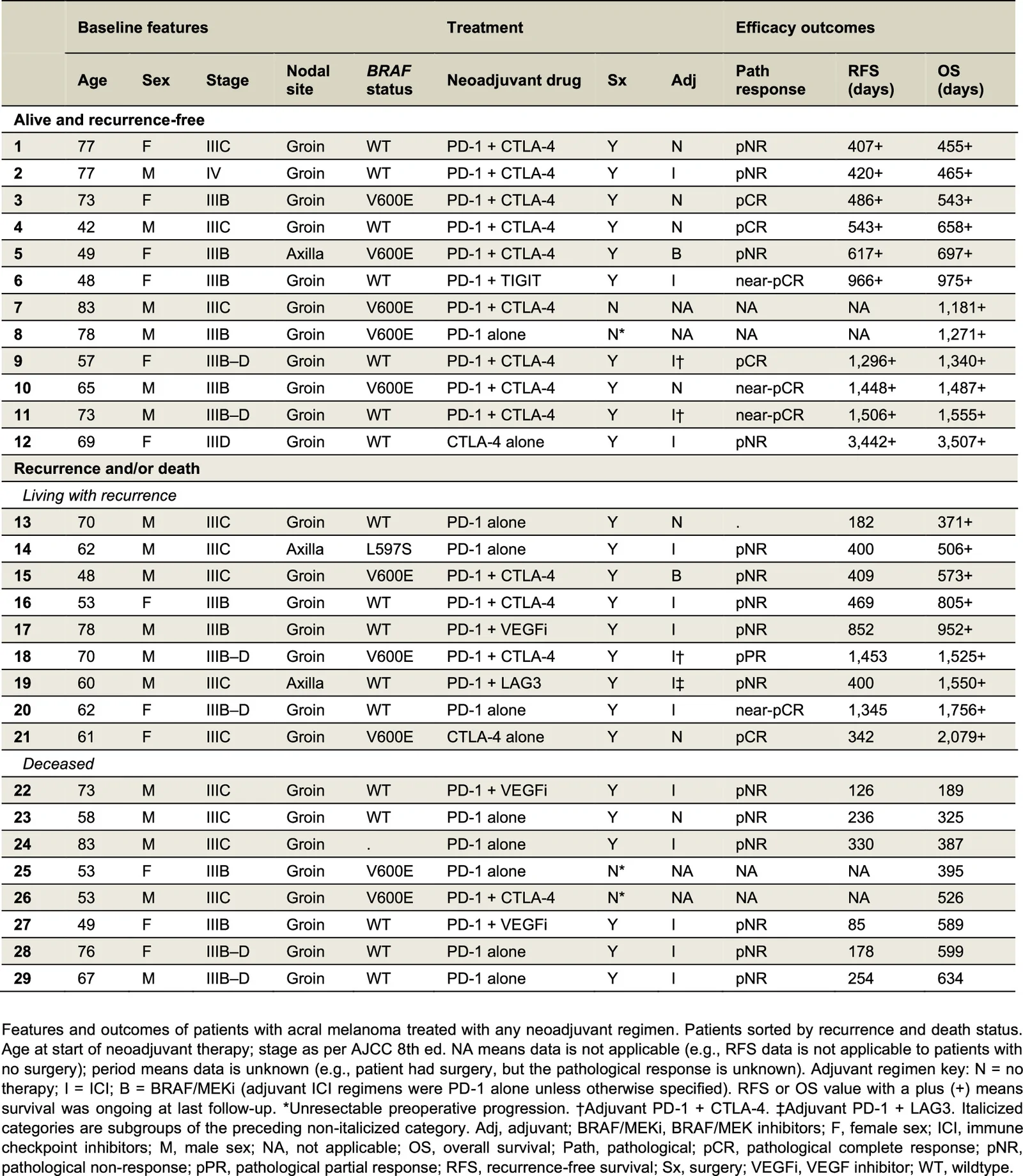
Features and outcomes of all patients with acral melanoma (N = 29)

**Extended Data Table 7:**
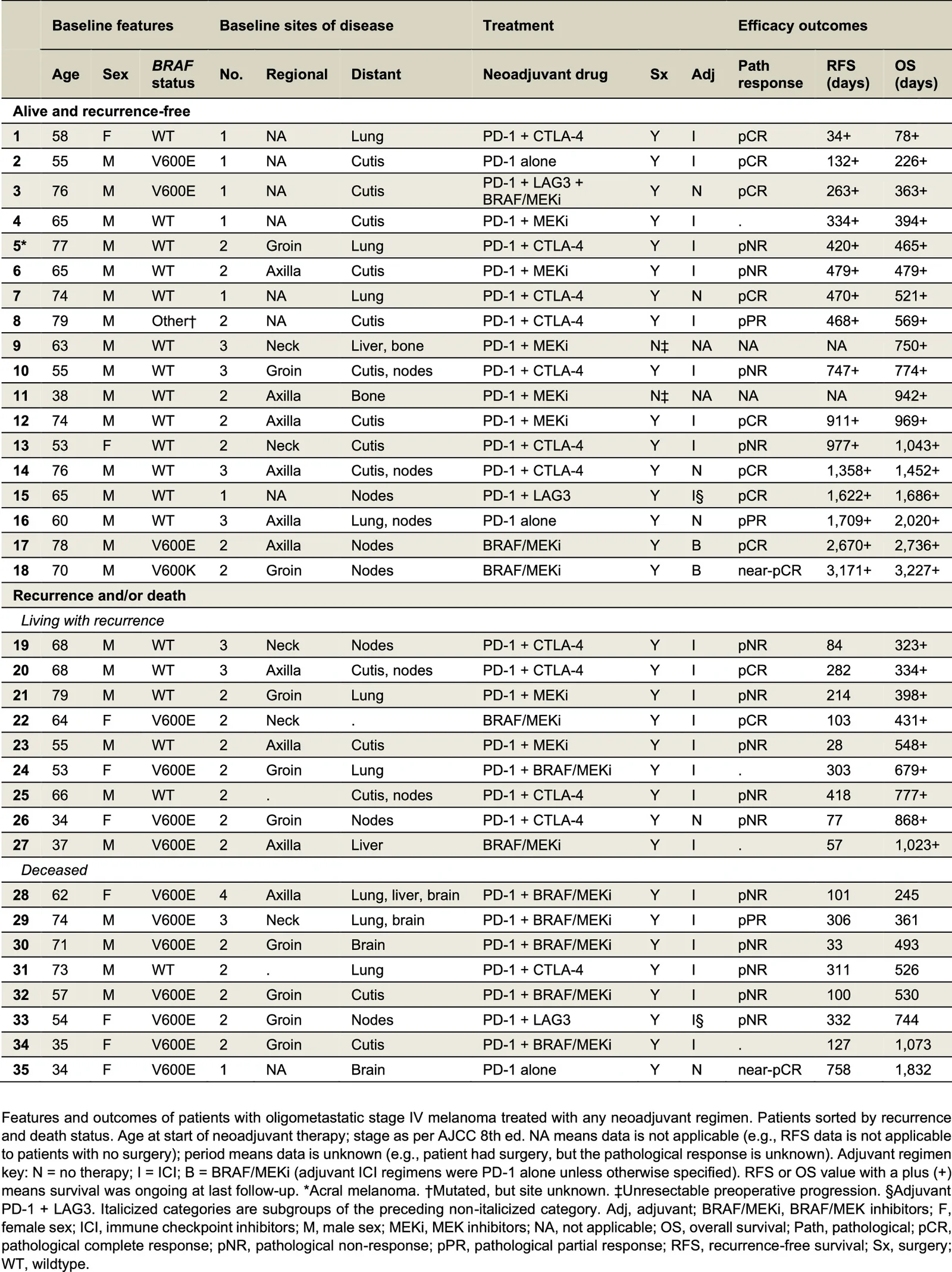
Features and outcomes of all patients with oligometastatic stage IV melanoma (N = 35)

## Data Availability

This multicenter clinical dataset is not freely available due to data-sharing agreements between the lead authors and several participating institutions. Requests for access will be reviewed by the lead authors and discussed with the relevant centers per existing agreements. Applicants can expect a response within 2 weeks of submission. Deidentified data will be provided under the following conditions: (1) the research must have received ethical approval from a recognized ethics review board; (2) the request must align with the scientific aims and goals of the dataset; (3) the requesting team must demonstrate the ability to handle the data securely and responsibly; and (4) a formal data usage agreement must be signed, ensuring that the data will not be used for commercial purposes and will not be shared with unauthorized parties.
