# Supplementary material for: Five-year survival with neoadjuvant therapy in melanoma: updated pooled analysis from the International Neoadjuvant Melanoma Consortium (INMC)
Source: Nat Med. 2026 Oct 1;32(11):4136–47. doi: 10.1038/s41591-026-04677-z (PMC13768178; doi:10.1038/s41591-026-04677-z)
Supplement: Supplementary file 1 [file 41591_2026_4677_MOESM1_ESM.pdf]

nature medicine

Supplementary information https://doi.org/10.1038/s41591-026-04677-z

Five-year survival with neoadjuvant therapy in melanoma: updated pooled analysis from the International Neoadjuvant Melanoma Consortium (INMC)

In the format provided by the authors and unedited

Supplementary Information

Supplementary Results **2**
Supplementary Results 1 | Radiological and metabolic response 2

Supplementary Tables **3**
Supplementary Table 1 | Contributing sites (N=1,038) 3
Supplementary Table 2 | Neoadjuvant regimens (N=1,038) 4
Supplementary Table 3 | Clinical trials (N=1,038) 5
Supplementary Table 4 | Clinicopathological factors associated with pCR in patients treated with ICI (N=735) 6

Supplementary Figures **7**
Supplementary Fig. 1 | RFS and pathological response by CT and FDG-PET response in patients treated with ICI (N=735) 7
Supplementary Fig. 2 | Bubble plots of MPR rate versus 3-year RFS rate with MPR by treatment regimen (N=1,038) 8
Supplementary Fig. 3 | Alluvial plot of imaging responses and recurrence in patients treated with ICI (N=735) 9
Supplementary Fig. 4 | EFS, RFS, and OS by sex in patients treated with ICI (N=735) 10

Supplementary References **11**

Supplementary Results

Supplementary Results 1 | Radiological and metabolic response

Radiological (CT; RECIST version 1.1[1]) and metabolic (FDG-PET; EORTC categories[2]) responses are shown in **Extended Data Table 5**. The objective response rate as per RECIST 1.1 was 49.8% (95% CI 45.8–53.8) with ICI, 83.0% (95% CI 75.0–89.3) with BRAF/MEKi, and 54.7% (95% CI 47.2–62.2) with ICI + TT, with progressive disease (PD) rates of 16.8%, 0.8%, and 8.4%, respectively. On FDG-PET, the objective metabolic response rate was 63.6% (95% CI 56.2–70.5) with ICI, 85.7% (95% CI 57.2–98.2) with BRAF/MEKi, and 75.2% (95% CI 65.7–83.3) with ICI + TT, and progressive metabolic disease (PMD) rates were 22.8%, 0%, and 7.9%, respectively. Pathological response and RFS rates by imaging response category are shown in **Supplementary Fig. 1**.

In 121 ICI-treated patients with available percent change from baseline data per CT (sum-of-diameters) and FDG-PET (SUVmax), plus recurrence data, we found that CT alone, PET alone, and CT plus PET models had similar, modest ability to predict recurrence at 1 year (AUC of 0.54, 0.49, and 0.54, respectively; p=0.821), 2 years (AUC of 0.69, 0.73, and 0.72; p=0.964), and 3 years (AUC of 0.68, 0.72, and 0.69; p=0.246). **Supplementary Fig. 3** contains an alluvial plot visualizing the relationship between complete imaging response on CT and/or FDG-PET and recurrence in patients treated with ICI.

Of the 308 ICI-treated patients with an objective CT response, surgery was omitted in 12 (3.9%). One 86-year-old male, who received neoadjuvant anti-CTLA-4 10 mg/kg, died due to melanoma after 7.7 years. The remaining 11 (91.7%) patients were alive and recurrence-free.

Supplementary Table 1 | Contributing sites (N=1,038)

	Trial (N=755)	Non-trial (N=283)	Overall (N=1,038)
**≥10% of patients**			
Melanoma Institute Australia, Australia	288 (38.1)	15 (5.3)	303 (29.2)
Netherlands Cancer Institute, The Netherlands	143 (18.9)	0 (0)	143 (13.8)
**≥5% and <10% of patients**			
National Tumour Institute Pascale Foundation, Italy	94 (12.4)	0 (0)	94 (9.1)
MD Anderson Cancer Center, TX, USA	92 (12.2)	0 (0)	92 (8.9)
Mayo Clinic Comprehensive Cancer Center, MN, USA	19 (2.5)	44 (15.6)	63 (6.1)
Gustave Roussy, France	27 (3.6)	25 (8.9)	52 (5.0)
**<5% of patients**			
Ludwig Maximilian University Hospital, Germany	0 (0)	35 (12.4)	35 (3.4)
Peter MacCallum Cancer Centre, Australia	12 (1.6)	23 (8.1)	35 (3.4)
AC Camargo Cancer Center, Brazil	0 (0)	28 (9.9)	28 (2.7)
University of Pennsylvania, PA, USA	26 (3.4)	0 (0)	26 (2.5)
Moffitt Cancer Center, FL, USA	0 (0)	21 (7.4)	21 (2.0)
N. N. Blokhin National Medical Research Centre of Oncology, Russia	0 (0)	21 (7.4)	21 (2.0)
University Hospital Tübingen, Germany	0 (0)	19 (6.7)	19 (1.8)
Saint-Louis Hospital, France	4 (0.5)	14 (5.0)	18 (1.7)
Maria Sklodowska-Curie National Research Institute of Oncology, Poland	0 (0)	13 (4.6)	13 (1.3)
Vanderbilt University Medical Center, TN, USA	0 (0)	14 (5.0)	14 (1.3)
Vall Hebron University Hospital, Spain	2 (0.3)	8 (2.8)	10 (1.0)
The Angeles Clinic and Research Institute, LA, USA	6 (0.8)	3 (1.1)	9 (0.9)
Blacktown Hospital, Australia	8 (1.1)	0 (0)	8 (0.8)
University Medical Center Groningen, The Netherlands	8 (1.1)	0 (0)	8 (0.8)
European Institute of Oncology Multimedica Group, Italy	6 (0.8)	0 (0)	6 (0.6)
Memorial Sloan Kettering Cancer Center, NY, USA	6 (0.8)	0 (0)	6 (0.6)
Leiden University Medical Center, The Netherlands	5 (0.7)	0 (0)	5 (0.5)
Erasmus Medical Center, The Netherlands	4 (0.5)	0 (0)	4 (0.4)
Utrecht Medical Center, The Netherlands	3 (0.4)	0 (0)	3 (0.3)
ESweden, Sweden	2 (0.3)	0 (0)	2 (0.2)

Final included patients per site.

Supplementary Table 2 | Neoadjuvant regimens (N=1,038)

	Abbreviation for Results, Tables, and Figs.	Trial (N=755)	Non-trial (N=283)	Overall (N=1,038)
**ICI*, n (%)**				
Anti-CTLA-4 alone	CTLA-4 alone	0 (0)	4 (1.4)	4 (0.4)
Anti-PD-1 alone	PD-1 alone	87 (11.5)	155 (54.8)	242 (23.3)
Anti-PD-1 + IO†	PD-1 + IO	376 (49.8)	113 (39.9)	489 (47.1)
Anti-PD-1 + anti-CTLA-4	PD-1 + CTLA-4	275 (36.4)	111 (39.2)	386 (37.2)
Anti-PD-1 + anti-LAG3	PD-1 + LAG3	56 (7.4)	2 (0.7)	58 (5.6)
Anti-PD-1 + other IO	PD-1 + other IO	45 (6.0)	0 (0)	45 (4.3)
**BRAF/MEKi, n (%)**				
BRAFi alone	BRAFi alone	0 (0)	1 (0.4)	1 (0.1)
BRAF/MEKi	BRAF/MEKi	112 (14.8)	6 (2.1)	118 (11.4)
**ICI + TT*, n (%)**	ICI + TT			
Anti-PD-1 + MEKi	PD-1 + MEKi	44 (5.8)	0 (0)	44 (4.2)
Anti-PD-1 + BRAF/MEKi	PD-1 + BRAF/MEKi	80 (10.6)	0 (0)	80 (7.7)
Anti-PD-1 + VEGFi	PD-1 + VEGFi	22 (2.9)	0 (0)	22 (2.1)
Anti-PD-1 + HDACi‡	PD-1 + HDACi	20 (2.6)	0 (0)	20 (1.9)
Anti-PD-1 + anti-CTLA-4 + HDACi‡	PD-1 + CTLA-4 + HDACi	14 (1.9)	0 (0)	14 (1.3)
Anti-PD-1 + anti-CTLA-4 + BRAF/MEKi	PD-1 + CTLA-4 + BRAF/MEKi	0 (0)	2 (0.7)	2 (0.2)
Anti-PD-1 + anti-LAG3 + BRAF/MEKi	PD-1 + LAG3 + BRAF/MEKi	0 (0)	2 (0.7)	2 (0.2)

Breakdown of neoadjuvant regimens included in the study by treatment setting (trial or non-trial). Each regimen and drug target includes a range of agents, dosing schedules, and treatment duration. Patients receiving intralesional therapies or chemotherapy were excluded. Italicized regimens are subsets of the preceding, but all percentages are calculated using the denominator in the column header. *PD-1 includes both anti-PD-1 and anti-PD-L1 agents. †IO includes any immuno-oncology agent(s), including other ICIs. ‡Patients receiving HDACi were from the DONIMI trial (NCT04133948) and stratified via IFNγ high or low. BRAF/MEKi, BRAF/MEK inhibitors; BRAFi, BRAF inhibitors; HDACi, HDAC inhibitors; ICI, immune checkpoint inhibitors; IFN, interferon; IO, immuno-oncology agent(s); MEKi, MEK inhibitors; TT, targeted therapy; VEGFi, VEGF inhibitors.

Supplementary Table 3 | Clinical trials (N=1,038)

	ICI (N=735)			BRAF/MEKi (N=119)	ICI + TT (N=184)	Overall (N=1,038)
	All (N=735)	PD-1 alone (N=242)	PD-1 + IO (N=489)			
**Non-trial patients, n (%)**	272 (37.0)	155 (64.0)	113 (23.1)	7 (5.9)	4 (2.2)	283 (27.3)
**Trial patients, n (%)**	463 (63.0)	87 (36.0)	376 (76.9)	112 (94.1)	180 (97.8)	755 (72.7)
PRADO[3]	99 (13.5)	0 (0)	99 (20.2)	0 (0)	0 (0)	99 (9.5)
NEO–TIM[4]	0 (0)	0 (0)	0 (0)	29 (24.4)	65 (35.3)	94 (9.1)
OpACIN–neo[5]	86 (11.7)	0 (0)	86 (17.6)	0 (0)	0 (0)	86 (8.3)
NeoTrio[6]	20 (2.7)	20 (8.3)	0 (0)	0 (0)	40 (21.7)	60 (5.8)
Morpheus–Melanoma[7]	57 (7.8)	1 (0.4)	56 (11.5)	0 (0)	0 (0)	57 (5.5)
NADINA[8]	52 (7.1)	0 (0)	52 (10.6)	0 (0)	0 (0)	52 (5.0)
BMS CA209-291[9]	49 (6.7)	11 (4.5)	38 (7.8)	0 (0)	0 (0)	49 (4.7)
Combi–Neo[10]	0 (0)	0 (0)	0 (0)	49 (41.2)	0 (0)	49 (4.7)
DONIMI[11]	10 (1.4)	10 (4.1)	0 (0)	0 (0)	34 (18.5)	44 (4.2)
KEYMAKER–U02C[12]	41 (5.6)	17 (7.0)	24 (4.9)	0 (0)	0 (0)	41 (4.0)
NeoCombi[13]	0 (0)	0 (0)	0 (0)	34 (28.6)	0 (0)	34 (3.3)
UPenn study[14]	26 (3.5)	26 (10.7)	0 (0)	0 (0)	0 (0)	26 (2.5)
NeoPeLe[15]	0 (0)	0 (0)	0 (0)	0 (0)	22 (12.0)	22 (2.1)
NeoACTIVATE[16]	0 (0)	0 (0)	0 (0)	0 (0)	19 (10.3)	19 (1.8)
OpACIN[18]	10 (1.4)	0 (0)	10 (2.0)	0 (0)	0 (0)	10 (1.0)
IEO838[19]	6 (0.8)	0 (0)	6 (1.2)	0 (0)	0 (0)	6 (0.6)
IOB-032/PN-E40[17]	6 (0.8)	1 (0.4)	5 (1.0)	0 (0)	0 (0)	6 (0.6)
SWOG S1801[20]	1 (0.1)	1 (0.4)	0 (0)	0 (0)	0 (0)	1 (0.1)

Final included clinical trials. BRAF/MEKi, BRAF/MEK inhibitors; ICI, immune checkpoint inhibitors; IO, immuno-oncology agent(s); TT, targeted therapy.

Supplementary Table 4 | Clinicopathological factors associated with pCR in patients treated with ICI (N=735)

	Univariate		Multivariate*	
	OR (95% CI)	P-value	OR (95% CI)	P-value
**Age (years)**	1.01 (1.00, 1.02)	0.2062		
**Sex**				
Female	1	0.4984		
Male	1.11 (0.82, 1.52)			
**Primary site**				
Extremity	1	0.0098		
Trunk	0.73 (0.47, 1.14)			
Head and neck	1.50 (0.92, 2.46)			
Acral	0.26 (0.08, 0.80)			
Occult	0.91 (0.58, 1.41)			
***BRAF* mutation status**				
Wildtype	1	0.0368	1	0.0438
V600	0.67 (0.48, 0.93)		0.67 (0.48, 0.93)	
Non-V600	1.04 (0.47, 2.30)		1.03 (0.45, 2.36)	
**Baseline disease**				
Nodal only	1	0.6401	1	0.0005
In-transits only	0.99 (0.50, 1.93)		0.52 (0.35, 0.78)	
Nodal plus in-transits	0.75 (0.41, 1.37)		0.44 (0.28, 0.68)	
**Nodal site**				
Neck	1	0.0008		
Axilla	0.57 (0.39, 0.84)			
Groin	0.46 (0.30, 0.69)			
**AJCC 8th ed. stage**				
IIIB	1	0.0303		
IIIC	0.68 (0.49, 0.94)			
IIID	0.34 (0.10, 1.10)			
IV	0.46 (0.17, 1.28)			
**SOD largest nodes (mm)**	0.99 (0.98, 1.00)	0.0379		
**Neoadjuvant ICI treatment type**				
PD-1 alone	1	0.0272		
PD-1 + IO	1.45 (1.04, 2.02)			
**Neoadjuvant therapy duration (weeks)**	1.00 (0.99, 1.02)	0.6336		
**Treatment course**				
Completed	1	0.8662		
Ceased early	0.96 (0.58, 1.57)			
**Treatment setting**				
Non-trial	1	0.6538		
Trial	0.93 (0.68, 1.28)			

Analysis included 682 ICI-treated patients with an available pathological response assessment, of whom 319 had pCR events. Age is at start of neoadjuvant treatment. *Variable selection for the multivariate model was performed using the penalized Least Absolute Shrinkage and Selection Operator (LASSO) method. All p-values were calculated using two-sided Wald tests. AJCC, American Joint Committee on Cancer; ICI, immune checkpoint inhibitors; IO, immuno-oncology agent(s); OR, odds ratio; pCR, pathological complete response; SOD, sum-of-diameters.

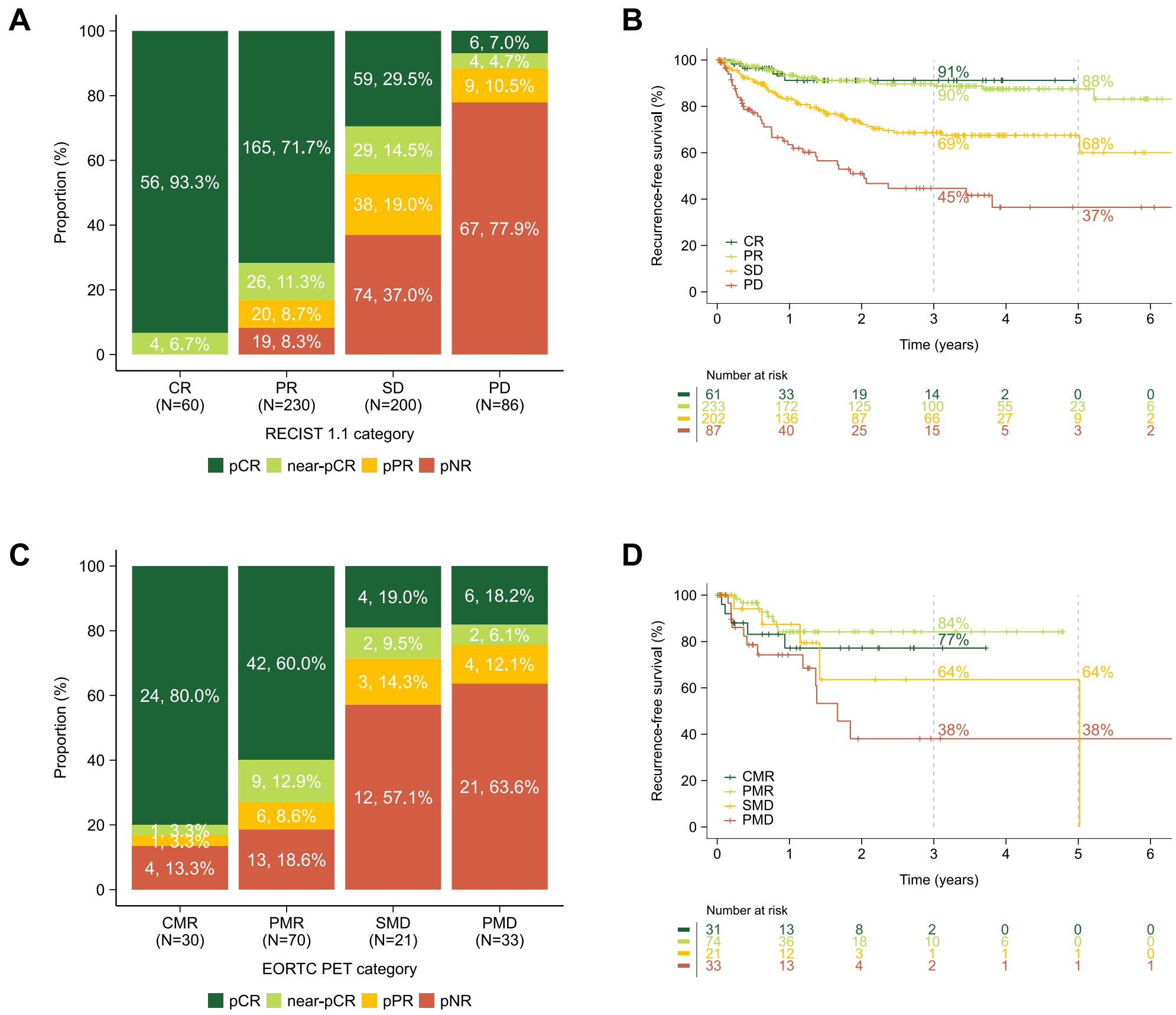

Supplementary Fig. 1 | RFS and pathological response by CT and FDG-PET response in patients treated with ICI (N=735)

Association of imaging response with pathological response and RFS in ICI-treated patients. Pathological response data shown in 100% stacked column graphs; white data labels are n, %. CT response as per RECIST 1.1[1] and FDG-PET response as per EORTC PET[2] categories. **A** See data labels. **B** Per RECIST 1.1: At 3 years, RFS rates were 91.2% (95% CI 83.2–100) with CR, 89.6% (85.3–94.2) with PR, 68.7% (61.7–76.5) with SD, and 44.6% (33.6–59.3) with PD. At 5 years, RFS rates were 87.5% (95% CI 82.4–93.0), 67.5% (60.3–75.6), and 36.5% (24.2–54.9) with PR, SD, and PD, respectively; 5-year data was not available for CR. **C** See data labels. **D** Per FDG-PET: At 3 years, RFS rates were 77.2% (95% CI 61.1–97.5) with CMR, 84.2% (74.6–95.0) with PMR, 63.6% (38.1–100) with SMD, and 38.1% (20.0–72.6) with PMD. At 5 years, RFS rates were 63.6% (95% CI 38.1–100) and 38.1% (20.0–72.6) with SMD and PMD, respectively; 5-year data was not available for CMR or PMR. CMR, complete metabolic response; CR, complete response; CT, computed tomography; FDG-PET, fluorodeoxyglucose positron emission tomography; ICI, immune checkpoint inhibitors; pCR, pathological complete response; PD, progressive disease; PMD, progressive metabolic disease; PMR, partial metabolic response; pNR, pathological non-response; pPR, pathological partial response; PR, partial response; RFS, recurrence-free survival; SD, stable disease; SMD, stable metabolic disease.

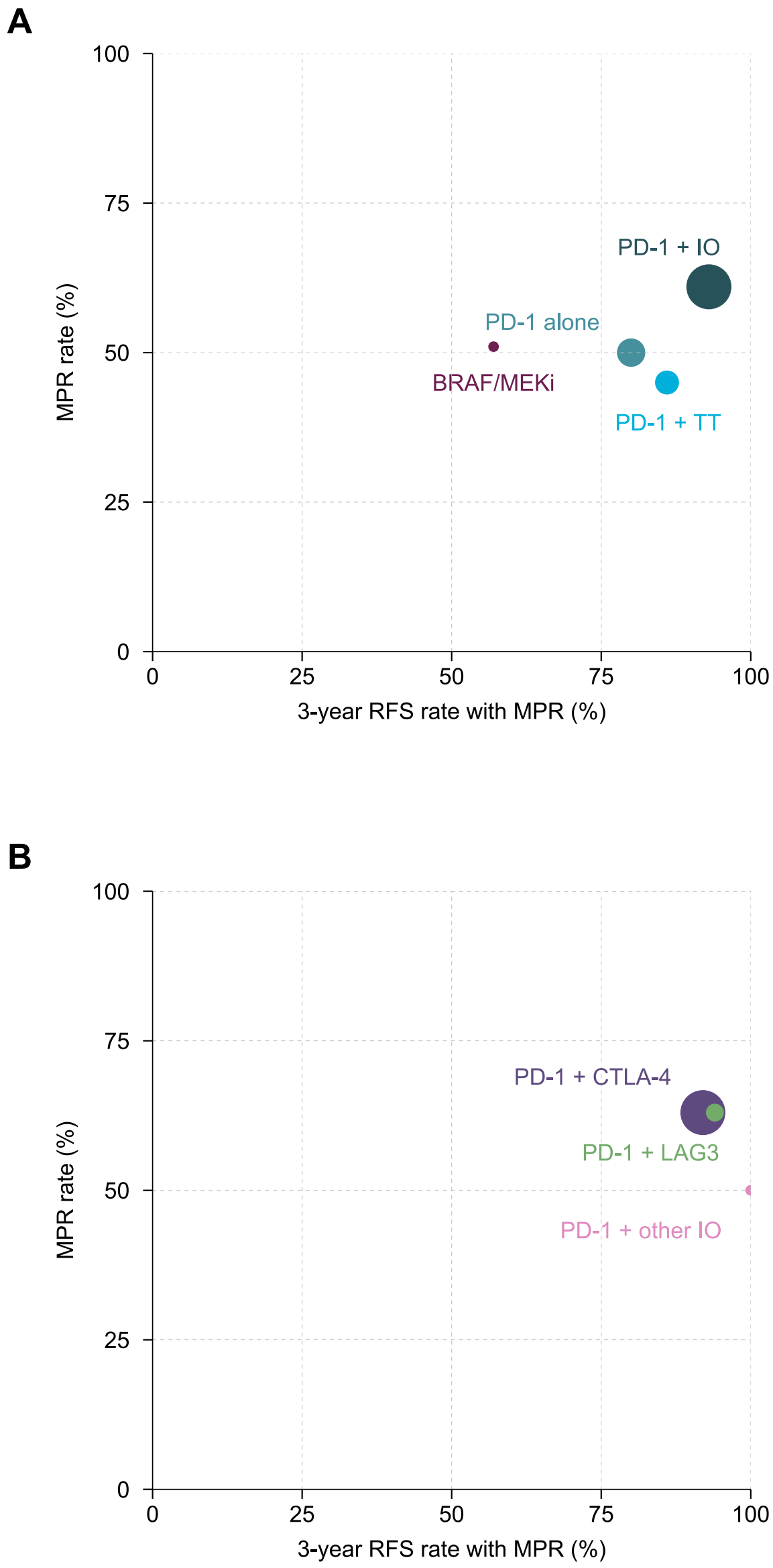

Supplementary Fig. 2 | Bubble plots of MPR rate versus 3-year RFS rate with MPR by treatment regimen (N=1,038)

Bubble plots of MPR rate (y-axis) and 3-year RFS rate in patients with MPR (x-axis) in 959 patients with pathological response data. Bubble size reflects number of patients. **A PD-1 alone:** MPR rate of 49.5% (103/208) and 3-year RFS rate with MPR of 95.6% (95% CI 89.2–100); **PD-1 + IO:** MPR rate of 61.4% (290/472) and 3-year RFS rate with MPR of 92.8% (89.7–96.1); **BRAF/MEKi:** MPR rate of 51.4% (57/111) and 3-year RFS rate with MPR of 60.4% (48.4–75.5); **ICI + TT:** MPR rate of 44.6% (74/166) and 3-year RFS rate with MPR of 89.6% (81.5–98.5). **B PD-1 + CTLA-4:** MPR rate of 62.5% (233/373) and 3-year RFS rate with MPR of 92.2% (88.6–96.0); **PD-1 + LAG3:** MPR rate of 63.2% (36/57) and 3-year RFS rate with MPR of 92.8% (83.4–100); **PD-1 + other IO:** MPR rate of 50.0% (21/42) and 3-year RFS rate with MPR of 100%. BRAF/MEKi, BRAF/MEK inhibitors; ICI, immune checkpoint inhibitors; IO, immuno-oncology agent/s; MPR, major pathological response; RFS, recurrence-free survival; TT, targeted therapy.

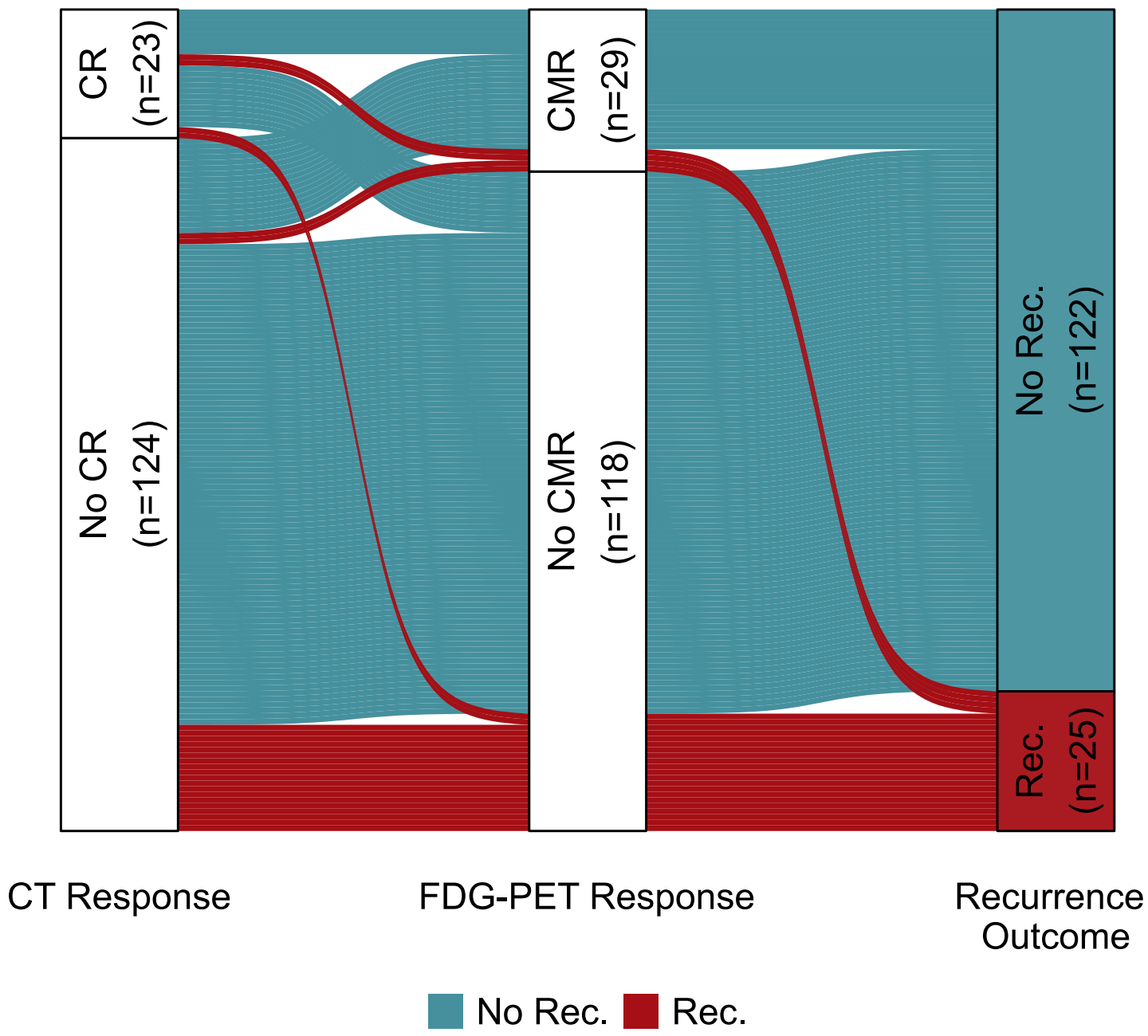

Supplementary Fig. 3 | Alluvial plot of imaging responses and recurrence in patients treated with ICI (N=735)

Alluvial plot of 147 ICI-treated patients with CT and FDG-PET response categories, plus recurrence data (including some patients where precise change-from-baseline is unknown). CT response as per RECIST 1.1[1] and PET-FDG response as per EORTC PET[2] categories. CMR, complete metabolic response on FDG-PET; CR, complete response on CT; CT, computed tomography; EORTC, European Organization for Research and Treatment of Cancer; FDG-PET, fluorodeoxyglucose positron emission tomography; ICI, immune checkpoint inhibitors; Rec., recurrence; RECIST, Response Evaluation Criteria in Solid Tumors.

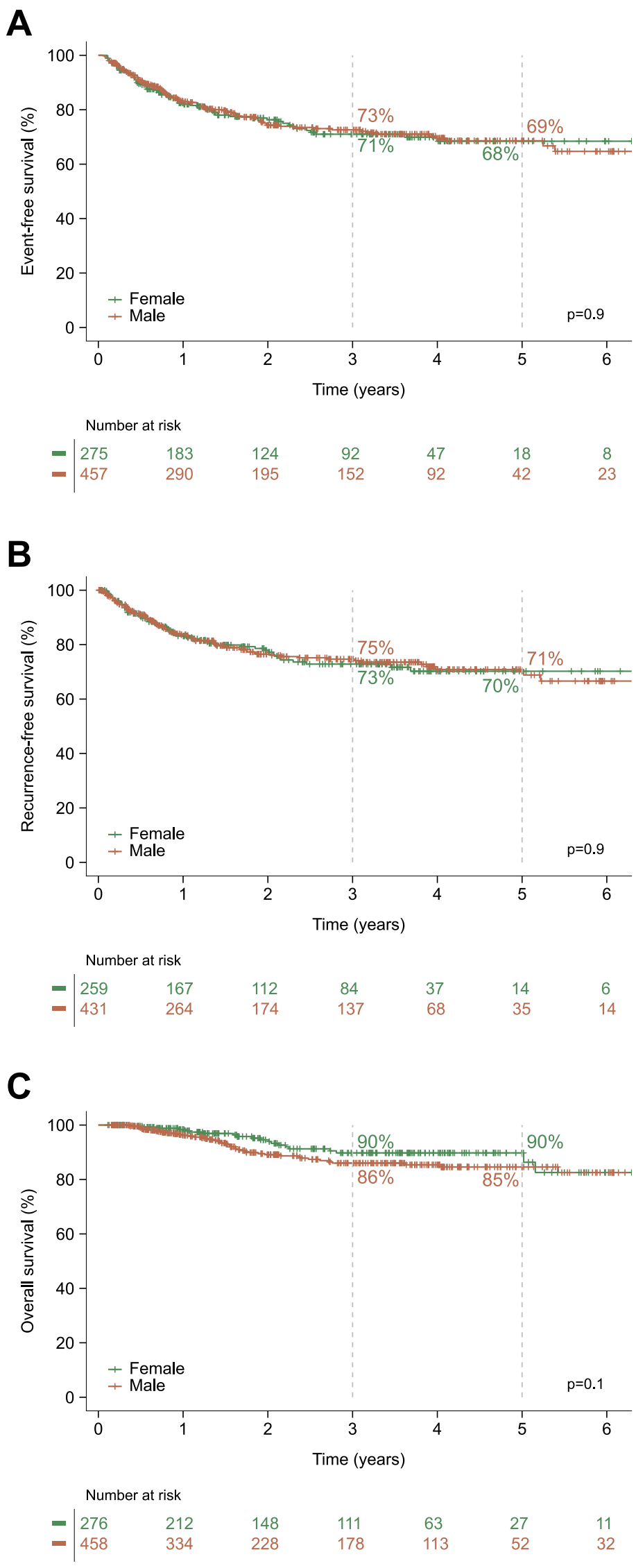

Supplementary Fig. 4 | EFS, RFS, and OS by sex in patients treated with ICI (N=735)

Survival rates for patients of female sex versus male sex treated with neoadjuvant ICI. **A** At 3 years, EFS rates were 71.0% (95% CI 65.1–77.5) in females and 72.6% (68.0–77.5) in males. At 5 years, EFS rates were 68.4% (95% CI 61.8–75.8) and 68.6% (63.3–74.4), respectively. **B** At 3 years, RFS rates were 72.8% (95% CI 66.7–79.5) in females and 74.7% (70.0–79.6) in males. At 5 years, RFS rates were 70.2% (95% CI 63.4–77.7) and 70.9% (65.4–76.8), respectively. **C** At 3 years, OS rates were 89.8% (95% CI 85.3–94.5) in females and 86.0% (82.1–90.1) in males. At 5 years, OS rates were 89.8% (95% CI 85.3–94.5) and 84.6% (80.3–89.1), respectively. EFS, event-free survival; ICI, immune checkpoint inhibitors; OS, overall survival; RFS, recurrence-free survival.

Supplementary References

1. Eisenhauer, E.A., *et al.* New response evaluation criteria in solid tumours: Revised RECIST guideline (version 1.1). *Eur. J. Cancer* **45**, 228–247 (2009).
2. Young, H., *et al.* Measurement of clinical and subclinical tumour response using [18F]-fluorodeoxyglucose and positron emission tomography: Review and 1999 EORTC recommendations. European Organization for Research and Treatment of Cancer (EORTC) PET study group. *Eur. J. Cancer* **35**, 1773–1782 (1999).
3. Reijers, I.L.M., *et al.* Personalized response-directed surgery and adjuvant therapy after neoadjuvant ipilimumab and nivolumab in high-risk stage III melanoma: The PRADO trial. *Nat. Med.* **28**, 1178–1188 (2022).
4. Ascierto, P.A., *et al.* Neoadjuvant plus adjuvant combined or sequenced vemurafenib, cobimetinib, and atezolizumab in patients with high-risk, resectable *BRAF*-mutated and wild-type melanoma: NEO–TIM, a phase II randomized non-comparative study. *Front. Oncol.* **13**, 1107307 (2023).
5. Rozeman, E.A., *et al.* Identification of the optimal combination dosing schedule of neoadjuvant ipilimumab plus nivolumab in macroscopic stage III melanoma (OpACIN−neo): A multicentre, phase 2, randomised, controlled trial. *Lancet Oncol.* **20**, 948–960 (2019).
6. Long, G.V., *et al.* Neoadjuvant pembrolizumab, dabrafenib and trametinib in *BRAF*(V600)-mutant resectable melanoma: The randomized phase 2 NeoTrio trial. *Nat. Med.* **30**, 2540–2548 (2024).
7. Long, G.V., *et al.* Neoadjuvant PD-1 and LAG-3-targeting bispecific antibody and other immune checkpoint inhibitor combinations in resectable melanoma: The randomized phase 1b/2 Morpheus–Melanoma trial. *Nat. Med.* **31**, 3700–3712 (2025).
8. Blank, C.U., *et al.* Neoadjuvant nivolumab and ipilimumab in resectable stage III melanoma. *N. Engl. J. Med.* **391**, 1696–1708 (2024).
9. Amaria, R.N., *et al.* Neoadjuvant relatlimab and nivolumab in resectable melanoma. *Nature* **611**, 155–160 (2022).
10. Amaria, R.N., *et al.* Neoadjuvant plus adjuvant dabrafenib and trametinib versus standard of care in patients with high-risk, surgically resectable melanoma: A single-centre, open-label, randomised, phase 2 trial. *Lancet Oncol.* **19**, 181–193 (2018).
11. Reijers, I.L.M., *et al.* IFN-gamma signature enables selection of neoadjuvant treatment in patients with stage III melanoma. *J. Exp. Med.* **220** (2023).
12. Dummer, R., *et al.* Neoadjuvant anti-PD-1 alone or in combination with anti-TIGIT or an oncolytic virus in resectable stage IIIB–D melanoma: A phase 1/2 trial. *Nat. Med.* **31**, 144–151 (2025).
13. Long, G.V., *et al.* Neoadjuvant dabrafenib combined with trametinib for resectable, stage IIIB–C, *BRAF*(V600) mutation-positive melanoma (NeoCombi): A single-arm, open-label, single-centre, phase 2 trial. *Lancet Oncol.* **20**, 961–971 (2019).

14. Huang, A.C.*, et al.* A single dose of neoadjuvant PD-1 blockade predicts clinical outcomes in resectable melanoma. *Nat. Med.* **25**, 454–461 (2019).
15. Long, G.V.*, et al.* NeoPeLe: A phase II trial of neoadjuvant (NAT) pembrolizumab (pembro) combined with lenvatinib (lenva) in resectable stage III melanoma. Presented at *ESMO Annual Congress* 2022, abstract no. **793P** (Paris, France).
16. Hieken, T.J.*, et al.* Neoadjuvant cobimetinib and atezolizumab with or without vemurafenib for high-risk operable stage III melanoma: The phase II NeoACTIVATE trial. *Nat. Commun.* **15**, 1430 (2024).
17. Long, G.V.*, et al.* A randomized phase 2 trial of the IO102–IO103 (IDO and PD-L1) cancer vaccine plus pembrolizumab as neoadjuvant/adjuvant treatment of patients with solid tumors. Presented at *ASCO Annual Congress* 2024, abstract no. **TPS2701** (Chicago, IL).
18. Blank, C.U.*, et al.* Neoadjuvant versus adjuvant ipilimumab plus nivolumab in macroscopic stage III melanoma. *Nat. Med.* **24**, 1655–1661 (2018).
19. Cocorocchio, E.*, et al.* Primary ipilimumab/nivolumab immunotherapy followed by adjuvant nivolumab in locally advanced or oligometastatic melanoma: Preliminary results. Presented at *ESMO Annual Congress* 2020, abstract no. **1147P** (Virtual).
20. Patel, S.P.*, et al.* Neoadjuvant-adjuvant or adjuvant-only pembrolizumab in advanced melanoma. *N. Engl. J. Med.* **388**, 813–823 (2023).
