# Supplementary material for: Five-year survival with neoadjuvant therapy in melanoma: updated pooled analysis from the International Neoadjuvant Melanoma Consortium (INMC)
Source: Nat Med. 2026 Oct 1;32(11):4136–47. doi: 10.1038/s41591-026-04677-z (PMC13768178; doi:10.1038/s41591-026-04677-z)
Supplement: Supplementary file 2 [file 41591_2026_4677_MOESM2_ESM.pdf]

nature portfolio

Corresponding author(s): Georgina V Long
Last updated by author(s): Aug 20, 2026

Reporting Summary

Nature Portfolio wishes to improve the reproducibility of the work that we publish. This form provides structure for consistency and transparency in reporting. For further information on Nature Portfolio policies, see our Editorial Policies and the Editorial Policy Checklist.

Statistics

For all statistical analyses, confirm that the following items are present in the figure legend, table legend, main text, or Methods section.

n/a	Confirmed	
☐	☒	The exact sample size (*n*) for each experimental group/condition, given as a discrete number and unit of measurement
☒	☐	A statement on whether measurements were taken from distinct samples or whether the same sample was measured repeatedly
☐	☒	The statistical test(s) used AND whether they are one- or two-sided<br>*Only common tests should be described solely by name; describe more complex techniques in the Methods section.*
☐	☒	A description of all covariates tested
☐	☒	A description of any assumptions or corrections, such as tests of normality and adjustment for multiple comparisons
☐	☒	A full description of the statistical parameters including central tendency (e.g. means) or other basic estimates (e.g. regression coefficient) AND variation (e.g. standard deviation) or associated estimates of uncertainty (e.g. confidence intervals)
☐	☒	For null hypothesis testing, the test statistic (e.g. *F*, *t*, *r*) with confidence intervals, effect sizes, degrees of freedom and *P* value noted<br>*Give P values as exact values whenever suitable.*
☒	☐	For Bayesian analysis, information on the choice of priors and Markov chain Monte Carlo settings
☒	☐	For hierarchical and complex designs, identification of the appropriate level for tests and full reporting of outcomes
☐	☒	Estimates of effect sizes (e.g. Cohen's *d*, Pearson's *r*), indicating how they were calculated

Our web collection on statistics for biologists contains articles on many of the points above.

Software and code

Policy information about availability of computer code

Data collection	Data were collected by investigators at each site and centrally collated and stored in Microsoft Excel for Microsoft 365 MSO (version 2408 +16.0.17928.20114) 64-bit.
Data analysis	Statistical analyses were performed using R (version 4.6.1 "Happy Hop") with RStudio (version 2025.06.0+242 "Blue Plumbago" Release (d1dc28bb, 2026-06-18) for Windows 64-bit). R packages included: dplyr (version 1.2.1), tidyr (version 1.3.2), stringr (version 1.6.0), forcats (version 1.0.1), survival (version 3.8-9), timeROC (version 0.4.1), table1 (version 1.5.1), ggplot2 (version 4.0.3), survminer (version 0.5.2), ggrepel (version 0.9.8), ggtext (version 0.1.2), ggalluvial (version 0.12.6), and pacman (version 0.5.1). Analyses were also performed using SAS (version 9.4).

For manuscripts utilizing custom algorithms or software that are central to the research but not yet described in published literature, software must be made available to editors and reviewers. We strongly encourage code deposition in a community repository (e.g. GitHub). See the Nature Portfolio guidelines for submitting code & software for further information.

Data

Policy information about availability of data

All manuscripts must include a data availability statement. This statement should provide the following information, where applicable:

- Accession codes, unique identifiers, or web links for publicly available datasets
- A description of any restrictions on data availability
- For clinical datasets or third party data, please ensure that the statement adheres to our policy

This multi-center clinical dataset is not freely available due to data-sharing agreements between the lead authors and several participating institutions. Requests for access will be reviewed by the lead authors and discussed with relevant centers per existing agreements. Applicants can expect a response within 2 weeks of submission. De-identified data will be provided under the following conditions: (1) the research must have received ethical approval from a recognized ethics review board; (2) the request must align with the scientific aims and goals of the dataset; (3) the requesting team must demonstrate the ability to handle the data securely and responsibly; (4) a formal data usage agreement must be signed, ensuring that the data will not be used for commercial purposes and will not be shared with unauthorized parties.

Research involving human participants, their data, or biological material

Policy information about studies with human participants or human data. See also policy information about sex, gender (identity/presentation), and sexual orientation and race, ethnicity and racism.

Reporting on sex and gender	Participants of all sexes and genders were eligible. Biological sex was collected and reported in the manuscript: female sex, n=397; male sex, n=641; non-female/non-male sex, n=0. There was no reporting on gender identity or sexual orientation.
Reporting on race, ethnicity, or other socially relevant groupings	Data on race, ethnicity, or any other social grouping was not collected.
Population characteristics	Patients with surgically resectable, clinically detected American Joint Cancer Committee (AJCC) 8th edition stage ≥IIIB cutaneous and acral melanoma were eligible, including patients with nodal disease, in-transit disease, and/or distant metastases. All patients received systemic neoadjuvant therapy with an immune checkpoint inhibitor (ICI)-based immunotherapy, BRAF/MEK inhibitors (BRAF/MEKi), or ICI plus any targeted therapy (TT). Neoadjuvant chemotherapy and intralesional therapy were excluded. Patients were included irrespective of whether they underwent surgery. Surgeries could include resection of in-transit or satellite disease, therapeutic lymph node dissection (TLND), index node resection, and/or metastasectomy, and could be followed by adjuvant therapy or not. There was no restriction on previous anticancer treatments.<br><br>Participants (N=1,038) had a median age of 60 years (IQR 49–69) at the start of neoadjuvant treatment. Sex was male in 61.8% (n=641), female in 38.2% (n=397), and non-female/non-male in 0% (n=0). BRAF mutation status was wildtype in 42.0% (n=436), V600 mutation in 49.1% (n=510), and non-V600 mutation in 2.8% (n=29). AJCC 8th edition stage was III in 96.6% (n=1,003), including IIIB in 42.3% (n=439) and IIIC in 41.4% (n=430), and IV in 3.4% (n=35). Primary tumor site was extremity (non-acral) in 25.1% (n=261), acral in 2.8% (n=29), trunk in 21.5% (n=223), head and neck in 11.9% (n=124), and occult in 20.2% (n=210). Baseline disease was nodal only in 82.4% (n=855), in-transit metastases only in 7.1% (n=74), and nodal plus in-transit metastases in 7.1% (n=74). Neoadjuvant treatment was ICI in 70.8% (n=735), BRAF/MEKi in 11.5% (n=119), and ICI + TT in 17.7% (n=184). Adjuvant treatment was received by 71.7% (n=744) of participants, comprising ICI in 52.6% (n=546) and BRAF/MEKi in 13.6% (n=141).
Recruitment	Not applicable, as this was a retrospective study. The cohort included patients recruited to 18 clinical trials registered with clinicaltrials.gov and/or EudraCT (see Supplementary Table 2). The cohort also included non-trial patients treated at specialised cancer centres who consented to data use in research studies.
Ethics oversight	This study was endorsed by the Melanoma Institute Australia (MIA) Research Committee and approved through the Royal Prince Alfred Hospital Ethics Review Committee (HREC/11/RPAH/32) under protocol X15-0311 & 2019/ETH06854 (2019 through 2030). All sites obtained local relevant approvals and were provided with the MIA study protocol and data collection forms. Informed consent was obtained from all individual participants included in the study via the institutional general research registry at the time of admission. There was no participant compensation. All data were de-identified prior to analysis.

Note that full information on the approval of the study protocol must also be provided in the manuscript.

Field-specific reporting

Please select the one below that is the best fit for your research. If you are not sure, read the appropriate sections before making your selection.

☒ Life sciences ☐ Behavioural & social sciences ☐ Ecological, evolutionary & environmental sciences

For a reference copy of the document with all sections, see nature.com/documents/nr-reporting-summary-flat.pdf

Life sciences study design

All studies must disclose on these points even when the disclosure is negative.

Sample size	This retrospective study pooled data from 1,038 patients treated at 26 international sites in routine care or within a clinical trial. As this study was intended to be a comprehensive sampling of patients treated with neoadjuvant therapy in a broad range of settings and with a broad range of systemic treatments, we collected as many patients as feasible.
Data exclusions	Exclusion criteria included patients with uveal or mucosal melanoma, and patients who had received neoadjuvant intralesional therapies or chemotherapy. Patients with uveal and mucosal melanoma were excluded because they are rare populations with differing treatment priorities and disease courses than cutaneous (including acral) melanomas. They are less amenable to definitive surgery, have poor response to immunotherapy, and have different standard-of-care treatments. Intralesional therapies were excluded because of the different mechanism of administration and because they are only approved in some countries, and only for unresectable melanoma. Chemotherapies were excluded because melanoma is largely chemoresistant and they are rarely used, especially in the neoadjuvant setting, which is of curative intent.
Replication	There was no replication involved. All patients were analysed as a single cohort.
Randomization	There was no randomization in this study. Patients were grouped and analysed by treatment received prior to the study.
Blinding	Investigators were not blinded to group allocation during data collection and analysis. As this was a retrospective study, outcomes were objective and pre-recorded, with group assignment inherent within the data structure, as opposed to a prospective, blinded clinical trial. Due to feasibility issues, there was no central or blinded review of histopathology or imaging data.

Reporting for specific materials, systems and methods

We require information from authors about some types of materials, experimental systems and methods used in many studies. Here, indicate whether each material, system or method listed is relevant to your study. If you are not sure if a list item applies to your research, read the appropriate section before selecting a response.

Materials & experimental systems

n/a	Involved in the study	
☒	☐	Antibodies
☒	☐	Eukaryotic cell lines
☒	☐	Palaeontology and archaeology
☒	☐	Animals and other organisms
☐	☒	Clinical data
☒	☐	Dual use research of concern
☒	☐	Plants

Methods

n/a	Involved in the study	
☒	☐	ChIP-seq
☒	☐	Flow cytometry
☒	☐	MRI-based neuroimaging

Clinical data

Policy information about clinical studies

All manuscripts should comply with the ICMJE guidelines for publication of clinical research and a completed CONSORT checklist must be included with all submissions.

Clinical trial registration	This was a retrospective, multicenter, international pooled analysis. As it was not a prospective clinical study, it was not eligible for registration. Patients from 18 trials (all registered) were included in the study: PRADO (NCT02977052) NEO–TIM (EUDRA2018-004841-17), OpACIN–neo (NCT02977052), NeoTrio (NCT02858921), Morpheus–Melanoma (NCT05116202), NADINA (NCT04949113), BMS CA209-291 (NCT02519322), Combi–Neo (NCT02231775), DONIMI (NCT04133948), KEYMAKER–U02C (NCT04303169), NeoCombi (NCT01972347), UPenn study (NCT02434354), NeoPeLe (NCT04207086), NeoACTIVATE (NCT03554083), IOB-032/PN-E4015 (NCT05280314), OpACIN (NCT02437279), IEO83817 (EUDRA2018-002172-40), and SWOG S180118 (NCT03698019). These are listed in Supplementary Table 3.
Study protocol	There was no study protocol, as this was not a prospective study. Instead, each site was provided with a 3-page study synopsis that explained the Background/Rationale, Study Aims and Objectives, Study Design, Study Population, Outcomes and Measures, Statistical Considerations, Abbreviations, and References. Once an investigator had agreed to participate, the investigator was sent a comprehensive data sheet (Microsoft Excel; see 'Data Collection', above) to return completed to the lead author for data cleaning and analysis.
Data collection	From Nov 16, 2023, to Oct 29, 2024, data from 1,038 patients with cutaneous (including acral) clinically-detected resectable stage IIIB–IV melanoma treated with neoadjuvant systemic therapy was collected from 26 international cancer research centres and university teaching hospitals (Supplementary Table 1; first patient first dose Aug 9, 2011, and last patient first dose Aug 12, 2024). Collection sites included: Melanoma Institute Australia, Australia; Netherlands Cancer Institute, The Netherlands; National Tumour Institute Pascale Foundation, Italy; MD Anderson Cancer Center, TX, USA; Mayo Clinic Comprehensive Cancer Center, MN, USA; Gustave Roussy, France; Ludwig Maximilian University Hospital, Germany; Peter MacCallum Cancer Centre, Australia; AC Camargo Cancer Cener, Brazil; University of Pennsylvania, PA, USA; Moffitt Cancer Center, FL, USA; N. N. Blokhin National Medical Research

Centre of Oncology, Russia; University Hospital Tübingen, Germany; Saint-Louis Hospital, France; Maria Sklodowska-Curie National Research Institute of Oncology, Poland; Vanderbilt University Medical Center, TN, USA; Vall Hebron University Hospital, Spain; The Angeles Clinic and Research Institute, LA, USA; Blacktown Hospital, Australia; University Medical Center Groningen, The Netherlands; European Institute of Oncology Multimedica Group, Italy; Memorial Sloan Kettering Cancer Center, NY, USA; Leiden University Medical Center, The Netherlands; Erasmus Medical Center, The Netherlands; Utrecht Medical Center, The Netherlands; ESweden, Sweden.

Outcomes

As this was a retrospective study, no primary or secondary outcomes were specified. Rather, the study followed the primary and secondary outcomes typical of a prospective clinical trial in the neoadjuvant setting. The key outcomes were pathological response, event-free survival, recurrence-free survival, overall survival, RECIST v1.1 response, and EORTC PET response. Pathological response was only included if scored per the original International Neoadjuvant Melanoma Consortium (INMC) criteria. Pathological response categories included pathological complete response (pCR; defined as the complete absence of residual viable tumour in the treated tumour bed), near-complete pCR (near-pCR; defined as >0% and ≤10% of the tumour bed occupied by viable tumour cells), pathological partial response (pPR; defined as >10% and ≤50% of the treated tumour bed occupied by viable tumour cells), and pathological non-response (pNR; defined as >50% of the tumour bed occupied by viable tumour cells. Major pathological response (MPR) included those with pCR or near-pCR (i.e., ≤10% viable tumour). EFS was defined as the time from the first dose of neoadjuvant therapy to unresectable pre-operative disease progression, recurrence, or death from any cause. RFS was defined as the time from surgery to recurrence or death from any cause. OS was defined as the time from first dose of neoadjuvant therapy to death from any cause. RECIST 1.1 response and PET EORTC response were calculated from CT scans and FDG-PET scans, respectively, at baseline and prior to surgery, in patients where data was available. Histopathology and imaging data were reviewed locally. Subgroup analyses included: adjuvant systemic therapy, BRAF mutation, in-transit metastases, distant metastases, acral melanoma, and treatment setting.

Plants

Seed stocks

NA

Novel plant genotypes

NA

Authentication

NA
